# Freshwater Conservation Priority Areas Are Threatened by Global Mining Activities

**DOI:** 10.1111/gcb.70774

**Published:** 2026-03-03

**Authors:** Mariana Braz Pires, Nina Baltus, Alexandra Marques, Rene Kleijn, Mike Buxton, Victor Maus, Valerio Barbarossa

**Affiliations:** ^1^ Institute of Environmental Sciences Leiden University Leiden the Netherlands; ^2^ Global Sustainability, PBL Netherlands Environmental Assessment Agency The Hague the Netherlands; ^3^ Geoscience and Engineering Department Delft University of Technology Delft the Netherlands; ^4^ Institute for Ecological Economics Vienna University of Economics and Business Vienna Austria; ^5^ Novel Data Ecosystems for Sustainability Group, Advancing Systems Analysis, International Institute for Applied Systems Analysis Laxenburg Austria

**Keywords:** coal, critical raw materials, energy transition, freshwater biodiversity, gold, mining, pollution, rivers

## Abstract

Mining poses significant and persistent threats to freshwater ecosystems, with impacts often enduring long after operations cease. Growing concerns suggest that the expansion of mining to meet global mineral demands for decarbonization may amplify these cumulative risks to freshwater biodiversity. However, the location and extent of potential conflict hotspots remain poorly understood, hampering our ability to meet international conservation targets. Here, we map areas of potential conflict between freshwater conservation priority areas and global mining activities. Using a spatial modeling approach, we trace potential downstream contamination and quantify the extent of affected river reaches within conservation priority areas. Our analysis reveals that mining may pollute up to 1.8 million km of downstream rivers (5% of the global total), over 18% of which lies within conservation priority areas. Gold mining is associated with the largest extent of potentially contaminated rivers, and its widespread reliance on unregulated small‐scale artisanal practices can lead to disproportionately severe impacts on freshwater biodiversity in affected areas. Furthermore, rivers potentially affected by coal mining far exceed those linked to the share of key energy transition minerals needed for clean energy technologies (cobalt, copper, graphite, lithium, nickel, and rare earth elements). Effectively safeguarding and restoring freshwater ecosystems will require conservation and regulatory frameworks that address downstream mining impacts, especially in the context of future mineral expansion.

## Graphical abstract

Meeting global mineral demands, including those for decarbonization, risks amplifying threats to freshwater biodiversity. This study presents the first global mapping of conflicts between mining and freshwater conservation, revealing that mining may contaminate up to 1.8 million km of rivers, with over 18% within conservation priority areas. Gold and coal mining are estimated to drive the largest potential impacts, far exceeding those from key energy transition minerals. These findings highlight critical gaps in current conservation and regulatory frameworks, calling for strategies that address downstream contamination from active and legacy mines to reconcile biodiversity preservation with mineral demands.

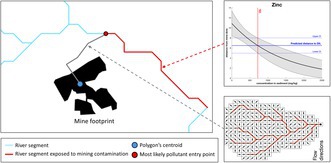

## 1 Introduction

Mining activities pose significant threats to biodiversity (Bennett 2016; Dudka and Adriano 1997; Durán et al. 2013; Giljum et al. 2022; Murguía et al. 2016; Siqueira‐Gay et al. 2020; Sonter et al. 2018), particularly in freshwater ecosystems (Lamb et al. 2024; Macklin et al. 2023), which rank among the most biodiverse yet threatened on the planet (Ahmed et al. 2022; Delong et al. 2023; Graça et al. 2024; Tockner et al. 2022). The resulting impacts often persist long after mining operations cease, especially where effective environmental regulations and rehabilitation measures are lacking (Atibu et al. 2016; Bennett 2016; Price and Wright 2016; Sonter et al. 2023; Wright and Burgin 2009). As the world transitions to a low‐carbon economy, shifting from coal to minerals essential for clean energy technologies (IEA 2022; Nijnens et al. 2023), cumulative impacts of mining on biodiversity are likely to increase over the coming decades, particularly if restoration measures are not significantly strengthened (Sonter et al. 2023), exacerbating challenges for conservation. This risk is further heightened by the fact that many of the minerals required for the energy transition are concentrated in biodiversity‐rich regions, placing some of the world's most ecologically valuable areas at greater risk (Luckeneder et al. 2021; Sonter et al. 2018, 2020). This directly conflicts with the Kunming‐Montreal Global Biodiversity Framework's commitment to protect 30% of the world's inland water ecosystems by 2030 (CBD 2022), demanding proactive policy responses to mitigate conflicts between mining and biodiversity conservation.

Freshwater ecosystems are particularly vulnerable to the diverse environmental pressures associated with mining, given their sensitivity to changes in water quality, hydrological processes, and habitat integrity (Barbarossa et al. 2026; Jain et al. 2016; Jones et al. 2018). Substantial amounts of mining‐derived pollutants enter surface waters, namely through acid mine drainage, altering water chemistry, increasing toxicant concentrations, and disrupting sediment and flow dynamics (Dethier et al. 2023; Hogsden and Harding 2012; Talukder et al. 2024). The effects of such contamination often extend far beyond extraction sites, as watercourses facilitate the downstream transport of sediments and hazardous metals over long distances (Bernhardt and Palmer 2011). In fact, metal mining alone is estimated to affect nearly 480,000 km of river channels globally (Macklin et al. 2023). The susceptibility of freshwater ecosystems is further reflected in their disproportionately high share of threatened species compared to terrestrial and marine systems (Mushtaq et al. 2022; Qin et al. 2024; Revenga et al. 2005). Many taxa, particularly macroinvertebrates, including key groups such as Ephemeroptera, Plecoptera, and Trichoptera, are highly sensitive to changes in water quality and hydrology, being widely used as bioindicators of ecosystem health (Albutra et al. 2017; Let et al. 2022; Marqués et al. 2003; Ngole‐Jeme and Ndava 2023). Moreover, mining‐related degradation has been shown to lead to pronounced shifts in macroinvertebrate community structure, as deteriorating conditions favor more tolerant groups and alter the composition of these assemblages (Bernhardt and Palmer 2011; Rico‐Sánchez et al. 2022). Fish populations are also severely affected, with declines and local extinctions reported in mining‐impacted areas (Affandi and Ishak 2019). Moreover, the accumulation of toxic metals in fish tissues not only poses risks to their populations and freshwater biodiversity, but also to human health, through processes of bioaccumulation and biomagnification (Gusso‐Choueri et al. 2018; Taiwo and Awomeso 2017; Zhu et al. 2015).

Protected Areas (PAs) and Key Biodiversity Areas (KBAs) are vital tools for the preservation of freshwater ecosystems. PAs provide legal protection for ecosystems and species, while KBAs identify critical sites for the global persistence of biodiversity, including freshwater habitats that support endangered and irreplaceable taxa (IUCN 2016; Moberg et al. 2024). Yet, freshwater ecosystems remain underrepresented in current conservation networks, with less than 15% of inland waters falling within designated PAs, well below global targets (CBD 2022), and with particularly low coverage in Africa and Asia (Bastin et al. 2019; Moberg et al. 2024). This shortfall is especially concerning because mineral deposits frequently occur in relatively intact and inaccessible regions, creating an inherent risk that new or expanding mining activities develop in areas with high conservation value. In fact, many mining operations already take place within or in close proximity to PAs and highly biodiverse and vulnerable regions (Luckeneder et al. 2021), potentially undermining ongoing conservation efforts and further challenging progress toward fulfilling international conservation commitments for freshwater ecosystems. Compounding this challenge, mining pressures can emerge abruptly as technological, economic, or political conditions shift, making their onset less predictable than other land‐use changes and hindering timely conservation responses.

Despite growing concerns about the impact of mining on freshwater ecosystems, the global effects of mining on freshwater biodiversity remain understudied. Most large‐scale assessments have primarily focused on terrestrial biodiversity (Murguía et al. 2016; Sonter et al. 2020). Sporadic research on freshwater impacts has typically either inferred risks from the IUCN threat classification and species geographic ranges, without explicitly modeling the hydrological propagation of contamination (Brabant et al. 2025; Lamb et al. 2024), or, when hydrological modeling of downstream contamination was undertaken (e.g., Macklin et al. (2023) for metal mining), it did not assess the potential implications for biological conservation. Consequently, the extent to which diverse mining‐related contamination may affect watercourses within areas of high conservation value for freshwater biodiversity remains unknown.

Here, we present the first global mapping of potential conflict areas between freshwater conservation priority sites (defined here as the combined extent of KBAs and PAs) and mining activities. We developed a spatial modeling approach integrating georeferenced global mining data with hydrological networks to trace potential downstream impacts and quantify affected river reaches within conservation priority sites. Our analysis employs a novel, harmonized dataset of mine locations and extents, significantly improving mining operations coverage compared to previous biodiversity assessments. With this study, we aim to provide spatially explicit evidence to inform conservation planning and guide policy decisions on mitigating mining‐related pressures on freshwater biodiversity.

## 2 Materials and Methods

### 2.1 Mining Data

Available global datasets on mining land‐use do not encompass every extraction site worldwide. However, multiple sources often provide complementary coverage, with areas of overlap. In this study, we used a global mining land‐use dataset (Maus 2026), which integrates three georeferenced mining land‐use datasets based on satellite image interpretation to achieve more comprehensive global coverage: Maus et al. (2022), Tang and Werner (2023), and OpenStreetMap. To supplement the data with information on mining commodities and other operational characteristics, Maus (2026) applied a hierarchical clustering algorithm based on geographic distance to associate land‐use polygons with mine‐property points from Jasansky et al. (2023), S&P Global Market Intelligence (2024), and Global Energy Monitor (2025), addressing cases where spatial proximity between features prevented direct attribution from available data or imagery.

The integrated dataset contains 217,201 polygons covering 150,643 km^2^, organized into 73,280 clusters, including both industrial and artisanal and small‐scale mining. Among the polygons, 48.1% contain information on the primary commodities mined (see detailed list of unique primary commodity fields in Table S1), covering 77.5% of the total mapped mining area. We identified mines extracting key energy transition minerals (ETMs), defined according to the latest International Energy Agency report on critical minerals (IEA 2024). The group of key ETMs included copper, lithium, nickel, cobalt, graphite, and rare earth elements. We also determined activity status at the polygon level using the established cluster correspondences, based on information available from the matched mine‐property points. As the original sources provided varying levels of detail, we harmonized reported statuses into four standardized categories: active, inactive, planned, and unknown (see Table S2). We excluded planned mines (842 polygons) from further analysis. In cases where multiple points within a cluster had conflicting status information, we applied a majority‐rule approach, assigning the most frequent status to all polygons in the cluster. When no single status predominated, we categorized the polygons in the cluster as uncertain (11,391 polygons); for analytical purposes, we grouped uncertain clusters with those classified as unknown (116,871 polygons; see Supporting Information Extended Methods and Tables S3–S5 for additional information on this category). The final dataset used in the analysis included 31,068 active and 57,029 inactive polygons.

### 2.2 Hydrological Data

To model the extent of rivers potentially exposed to mining‐related contamination, we retrieved hydrological data from the HydroRIVERS dataset (Linke et al. 2019), which provides a globally consistent, vector‐based representation of river networks. In addition, to link the mines to river reaches, we employed the flow direction raster layer from the HydroSHEDS database (Lehner et al. 2008) at a 15 arc‐second spatial resolution to maintain consistency with the spatial resolution of the hydrography used to generate the vector stream network.

### 2.3 Modeling Potential Downstream Exposure of Rivers to Mining

To identify river systems potentially exposed to mining‐related contamination, we developed a spatial modeling approach that, starting from the mine polygon, tracks downstream river segments until a commodity‐dependent threshold is reached. We implemented the model in R 4.2.2 and ArcGIS Pro 3.2.0.

#### 2.3.1 Linking Mines to Rivers

First, we linked the mines to the vectorized stream network to identify potential pollutant entry points into watercourses. This was done using two complementary methods: a centroid‐based approach and an intersection‐based approach (see Figure S1 for a schematic overview). In the centroid‐based method, we generated a point within each mine polygon using the ‘st_point_on_surface’ function from the R package ‘sf’ 1.0.16 (Pebesma 2018). While we refer to this point as a centroid for simplicity, it is guaranteed to lie within the polygon, avoiding cases where a geometric centroid might fall outside irregular shapes. These points were then connected to the nearest downstream watercourse by following flow paths through the function ‘taudem_moveoutletstostream’ from the R package ‘traudem’ 1.0.3 (Carraro et al. 2022). Input data included the centroids, the flow direction data, and a rasterized version of the HydroRIVERS stream network at a 15 arc‐second resolution to match the flow direction raster layer (see Section 2.2). To focus on areas where pollutants are most likely to enter a watercourse, we applied a 10 km buffer around all mines, excluding cases where the nearest downstream watercourse was beyond this threshold (Lin et al. 2022; Macklin et al. 2006). In total, 98.9% of the mine polygons were linked to a river, with a global average geodesic distance of 1.6 km and a standard deviation of 1.3 km.

While most mine polygons (87.3%) do not directly overlap with rivers, larger mining operations often encompass sections of a river within their footprint. To address this, we identified all river segments intersecting mine polygons and considered these segments directly exposed to mining‐related contamination. This intersection‐based approach complemented the previously described centroid‐based method by capturing cases where pollutants might be introduced into the river system from multiple points within a mine rather than a single entry.

To ensure the robustness of our linkage strategy, we implemented a randomized sampling approach, in which randomly selected points within each mine polygon were routed downslope to identify associated river segments. The results confirmed a high degree of agreement between methods; full methodological details and validation outcomes are provided in the SI (Extended Methods).

#### 2.3.2 Attenuation Distance Assumptions

Empirical studies linking mining activity to freshwater biodiversity remain limited, are often restricted to site‐level assessments, and rarely quantify biological responses along downstream gradients, which prevents identifying or validating a generalizable distance–response relationship (Sonter et al. 2018, 2023). As a result, to estimate the total length of river stretches potentially influenced by mining, we extended the identified exposure zones downstream using attenuation distances that represent the modeled dispersal of sediment‐associated metal contamination. Where available, we used the element‐specific attenuation distances reported by Macklin et al. (2023), who modeled thresholds at which metal concentrations fall below Dutch Intervention Limits. For commodities not included in Macklin et al. (2023), we implemented two modeling assumptions representing differing degrees of potential downstream contaminant spread: a moderate‐exposure and a severe‐exposure assumption. For brevity, we refer to these throughout as the “moderate” and “severe” assumptions, respectively. Under the moderate assumption, we assigned the shortest attenuation distance reported by Macklin et al. (6.5 km), while under the severe assumption we assigned the longest reported distance (45.6 km). Additionally, to account for inherent variability in mine and river characteristics not captured by commodity‐based distances, such as differences in extraction methods, pollution loads, and environmental conditions, we applied uniform thresholds of 6.5 km and 45.6 km across all commodities, representing lower‐bound and upper‐bound assumptions for downstream contamination (results provided in Supporting Information).

#### 2.3.3 Downstream Tracing Procedure

We implemented this analysis by developing a routine that traces potentially affected river segments downstream. For each mine, the routine starts at the mine‐linked river segments (for the centroid‐based approach) or the mine‐intersecting segments (for the intersection‐based approach). Using downstream connectivity information from the HydroRIVERS dataset (Linke et al. 2019), the model identifies consecutive river segments potentially influenced by mining until the predefined distance threshold is attained. For the centroid‐based approach, since mine‐linked entry points are not necessarily located at the start of a HydroRIVERS segment, we split each segment at the identified entry point using the ‘Split Line at Point’ tool in ArcGIS Pro, retaining only the downstream portion for potential impact estimation. Similarly, when the cumulative length of traced river segments approaches the attenuation threshold, the final segment is cropped to ensure that only the portion within the defined limit is included. For the intersection‐based approach, river segment portions within each mine polygon are treated as directly exposed and are included in the potentially affected network. Downstream propagation is then traced from the point where the river exits the mine polygon, accumulating the lengths of consecutively connected segments until the predefined attenuation distance is reached.

In both approaches, the tracing process is terminated if a lentic system (e.g., a lake or reservoir) is encountered in the downstream sequence. The dispersion dynamics of pollutants in lotic and lentic systems differ due to variations in water volume, flow characteristics, and mixing patterns. Because of these differences, lentic systems can act as sinks that disrupt downstream pollutant transport. To reflect this, when the model reaches a segment with limnicity greater than 20% (i.e., percent lake area (Messager et al. 2016)) in the downstream sequence, the tracing process is terminated at that point, ensuring that neither the segment itself nor those that follow it are included.

Some river segments were identified multiple times, due to overlapping exposure zones from different mines or the combined results of the centroid‐based and intersection‐based approaches. As such, we merged all overlapping river segments using the ‘Dissolve’ tool in ArcGIS Pro, ensuring that each affected river stretch was only represented once. We then calculated the geodesic distances of the unique potentially affected river segments using the function ‘st_length’ from R package ‘sf’ 1.0.16 (Pebesma 2018).

### 2.4 Overlap Between Potentially Impacted Rivers and Conservation Priority Areas

We retrieved shapefiles for the world's KBAs and PAs from publicly available datasets (BirdLife International 2023; UNEP‐WCMC and IUCN 2023), with updates from September 2023 for KBAs and November 2023 for PAs. Although KBAs and PAs are generally provided as mapped boundaries (polygons), some sites are represented as points when exact boundaries are unavailable. Where the site area was provided, we modeled these points as circular buffers matching the reported extent. Marine PAs were excluded from this procedure, as buffering large marine extents creates spatial uncertainty and risks erroneous terrestrial overlap. To address documented spatial coverage limitations and reporting biases in the WDPA, particularly for China, India, and Türkiye (UNEP‐WCMC and IUCN 2023), we supplemented the dataset with additional PA data from national sources (China Nature Reserve Specimen Resource Sharing Platform 2024; MOAF 2019; Wildlife Institute of India 2020). Our final KBA layer covered a total of 11,964,047 km^2^, while the PA layer covered 27,250,479 km^2^ of terrestrial and inland waters.

For this analysis, we defined conservation priority areas (CPAs) as the combined extent of all KBAs and PAs. Although other regions of conservation importance exist, including those identified in frameworks such as freshwater ecoregions (Abell et al. 2008) and biodiversity hotspots (Myers et al. 2000), they are based on broad biogeographic units rather than site‐based designations comparable to PAs or KBAs. We therefore focus our analysis on PAs and KBAs, as they represent globally recognized and spatially standardized site‐based conservation units suitable for consistent global assessments. We overlaid all river segments, including the ones potentially exposed to mining‐related contamination, with the CPA polygons, using the ‘st_intersection’ function from the ‘sf’ 1.0.16 R package (Pebesma 2018). We then calculated the geodesic length of the river segments located within these areas.

To account for differences in the expected enforcement of protection measures among WDPA designations, we repeated the full analytical workflow using a more conservative representation of PAs. In this case, KBAs were excluded, and the PA layer was restricted to nationally designated sites with an assigned IUCN management category (I–VI) and not classified as proposed, following Venter et al. (2014). The PAs obtained from national datasets were retained as provided, as comparable IUCN category and status information was not available. All spatial overlays and calculations were performed identically to those in the main analysis. This PA layer is hereafter referred to as IUCN‐categorized PAs.

We summarized the results by calculating both the total length and the proportion of potentially exposed river segments. The proportion was defined as the length of exposed river segments divided by the total river length within each unit. These values were computed at the global scale, and separately for each mining commodity (including the key ETMs' group), hydrological basin, KBA, and PA. For the commodity‐level analysis, river segments were grouped by the commodities associated with upstream mines. River segments affected by multiple commodities were counted under each relevant group, as this reflects the possibility of combined impacts from different types of mining activity. For the basin‐level analysis, HydroBASINS level 8 sub‐basins were aggregated based on their *MAIN_BAS* identifiers, which group all sub‐basins draining to the same most downstream sink within the level 8 hierarchy (Lehner and Grill 2013).

Recognizing that not all mined key ETMs are used in clean energy technologies, we estimated the proportion of potential impact attributable to clean energy demand by applying the IEA's projected 2023 demand shares for these minerals (IEA 2024). For 2023, the estimated demand percentages were 24.41% for copper, 55.76% for lithium, 15.40% for nickel, 29.77% for cobalt, 27.89% for graphite, and 17.20% for rare earth elements. These values were applied as global averages to contextualize the share of the potentially affected river length in CPAs linked to clean energy technologies. For mines extracting multiple ETMs as primary commodities, we averaged the demand percentages before scaling the potentially affected river lengths accordingly.

## 3 Results

**FIGURE 1:**
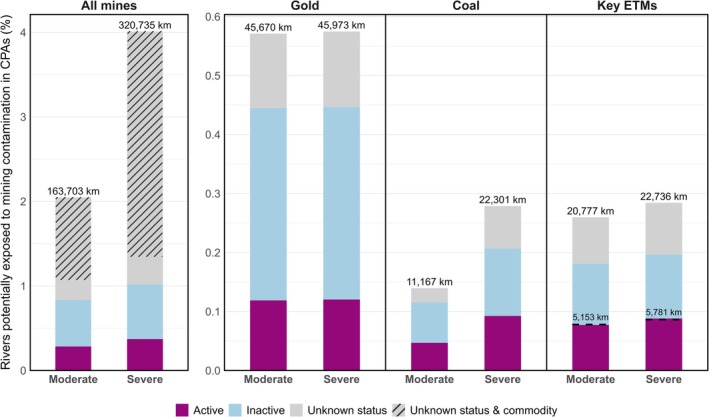
Proportion of global river length within conservation priority areas (CPAs) potentially exposed to mining‐related contamination. CPAs are defined as the combined extent of Protected Areas and Key Biodiversity Areas. The figure includes estimates under the moderate and severe modeling assumptions. Results are presented for all mines (left panel) and for three subsets of mines (right panels): Coal, gold, and key energy transition mineral (ETM) mines. Bar heights represent the percentage of river length within CPAs that is potentially impacted by mining activities, calculated as the ratio of potentially exposed river length within these areas to the total river length across all CPAs. Each bar separates the contributions of active, inactive, and mines with unknown activity status, including mines with unknown primary commodities (a subset of mines with unknown activity status). Absolute lengths of rivers potentially exposed to downstream mining contamination (km) are displayed on top of each bar. In the chart focused on key ETM mines, the area below the dashed black line indicates the estimated river length potentially exposed to contamination from mining of minerals used in clean energy technologies, based on 2023 demand estimates from IEA (2024). This portion includes all activity statuses, while the remaining bar segment reflects additional potential exposure from mining of these minerals for other uses.

Worldwide, between 1.0 and 1.8 million km of river networks (2.8%–5.1% of the global total) may be exposed to mining‐related contamination, under moderate and severe modeling assumptions, respectively (Dataset S1). A considerable share of these rivers, 16.9%–18.0%, is located within CPAs (13.2%–13.9% in PAs and 7.3%–8.1% in KBAs). This translates to mining activities potentially affecting 2.0%–4.0% of the total river length contained in CPAs (1.9%–3.7% in PAs and 2.3%–4.8% in KBAs; Figures 1 and S2). Within IUCN‐categorized PAs, approximately 81,000–150,000 km (8.4%–8.6%) of potentially impacted rivers are present (Figure S3).

**FIGURE 2:**
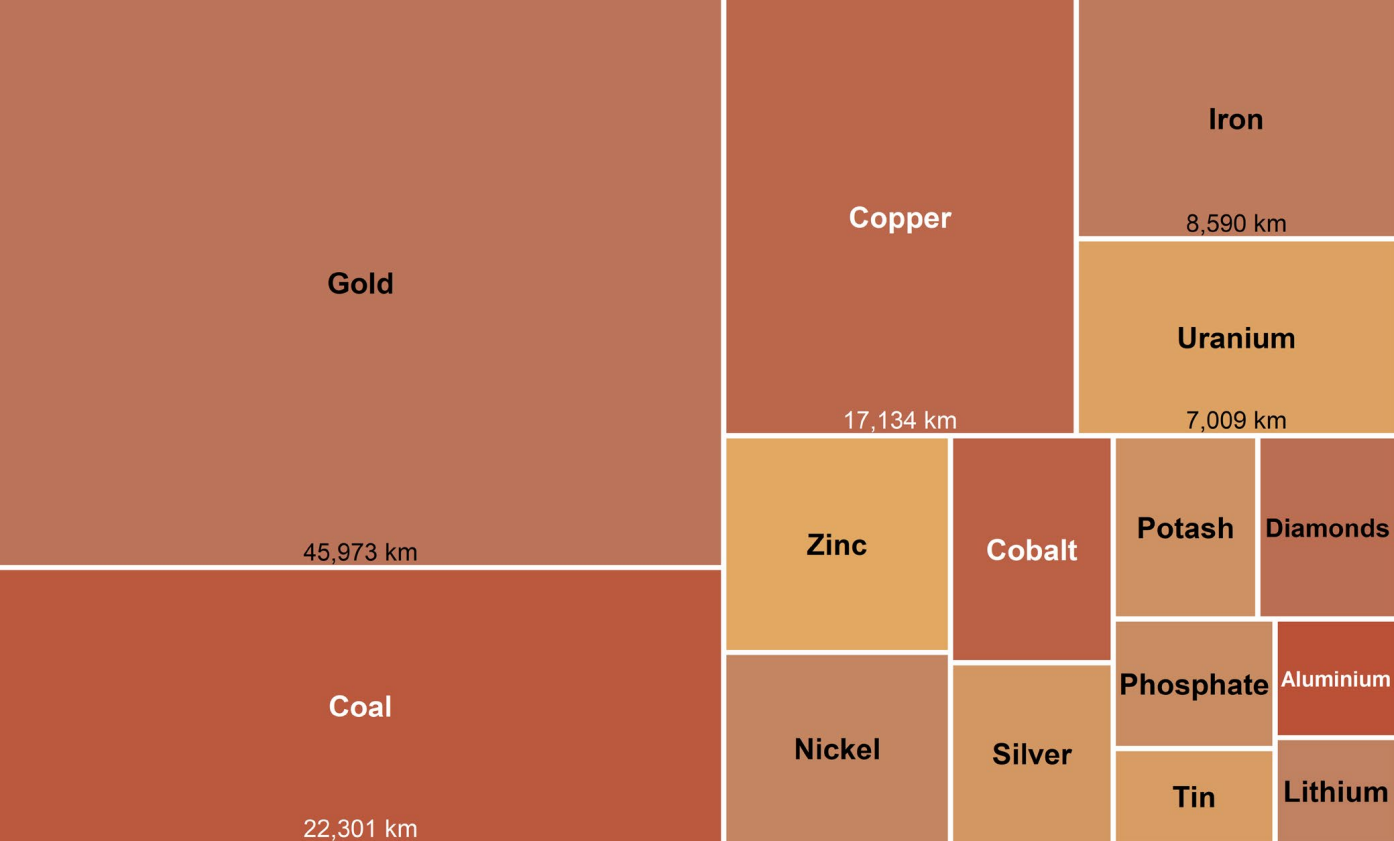
Relative contribution of primary mining commodities to the estimated global river length potentially exposed to mining‐related contamination within conservation priority areas (CPAs; i.e., the combined extent of Protected Areas and Key Biodiversity Areas). The figure displays the top 15 primary mining commodities with the highest estimated contribution based on the severe modeling assumption. Box sizes represent each commodity's share of the total potentially contaminated river length within CPAs. Because multiple mining commodities may influence a single river segment, values are not mutually exclusive. To improve clarity, the figure excludes the ‘Unknown’ category (representing 243,354 km of river length potentially influenced by mining), where data on the primary commodity of the mine were unavailable.

Gold, coal, and copper are the primary commodities associated with the greatest estimated river lengths potentially impacted by mining, a pattern that is consistent across global totals, CPAs, and IUCN‐categorized PAs (Figures 2 and S4–S9). Gold mining alone is estimated to influence nearly 275,000 km of global watercourses under the severe assumption, with corresponding values of approximately 165,000 km and 102,000 km for coal and copper, respectively (results under all modeling assumptions are provided in Supporting Information). When focusing on CPAs, gold mining is linked to approximately 46,000 km of potentially impacted rivers in CPAs, followed by coal (22,000 km) and copper (17,000 km). Considerable overlap remains within IUCN‐categorized PAs, where gold, coal, and copper are linked to approximately 26,000 km, 11,000 km, and 8200 km of potentially impacted rivers, respectively. Iron also ranks among the top commodities, with an estimated 62,000 km of potentially affected rivers globally and 8600 km within CPAs under the severe assumption. It should be noted that a large share of the potentially affected river network is associated with mines for which the primary commodity is unknown. Under the severe assumption, 1.26 million km of rivers (70.7% of all potentially affected rivers) are affected by a combination of known and unknown commodities, of which 1.08 million km (60.9%) are exclusively linked to mines with unknown commodities. Within CPAs, rivers associated with unknown commodity mines total 243,000 km, including 213,000 km (66.4%) exclusively linked to unknowns.

Collectively, key ETM mines are associated with an estimated 129,000–142,000 km of potentially affected rivers globally under the moderate and severe assumptions, respectively. Within CPAs, potentially affected river lengths associated with key ETM mines range from 21,000 km to 23,000 km, of which approximately 24.8%–25.4% is estimated to be linked to clean energy demand (Figure 1; Dataset S2). Within IUCN‐categorized PAs, key ETM mines are associated with nearly 10,000–11,000 km of rivers potentially exposed to mining pollution.

When comparing the potential influence of mines with known activity status, we found that currently active mines, which account for 14.4% of all mine polygons in the dataset, are linked to 163,000–216,000 km of potentially impacted river length (Dataset S1). Of this, approximately 23,000–30,000 km are located within CPAs (11,000–14,000 km within IUCN‐categorized PAs). On the other hand, inactive mines, representing 26.4% of the dataset, contribute 257,000–305,000 km of influenced river length, with approximately 44,000–52,000 km in CPAs (25,000–28,000 km in IUCN‐categorized PAs).

**FIGURE 3:**
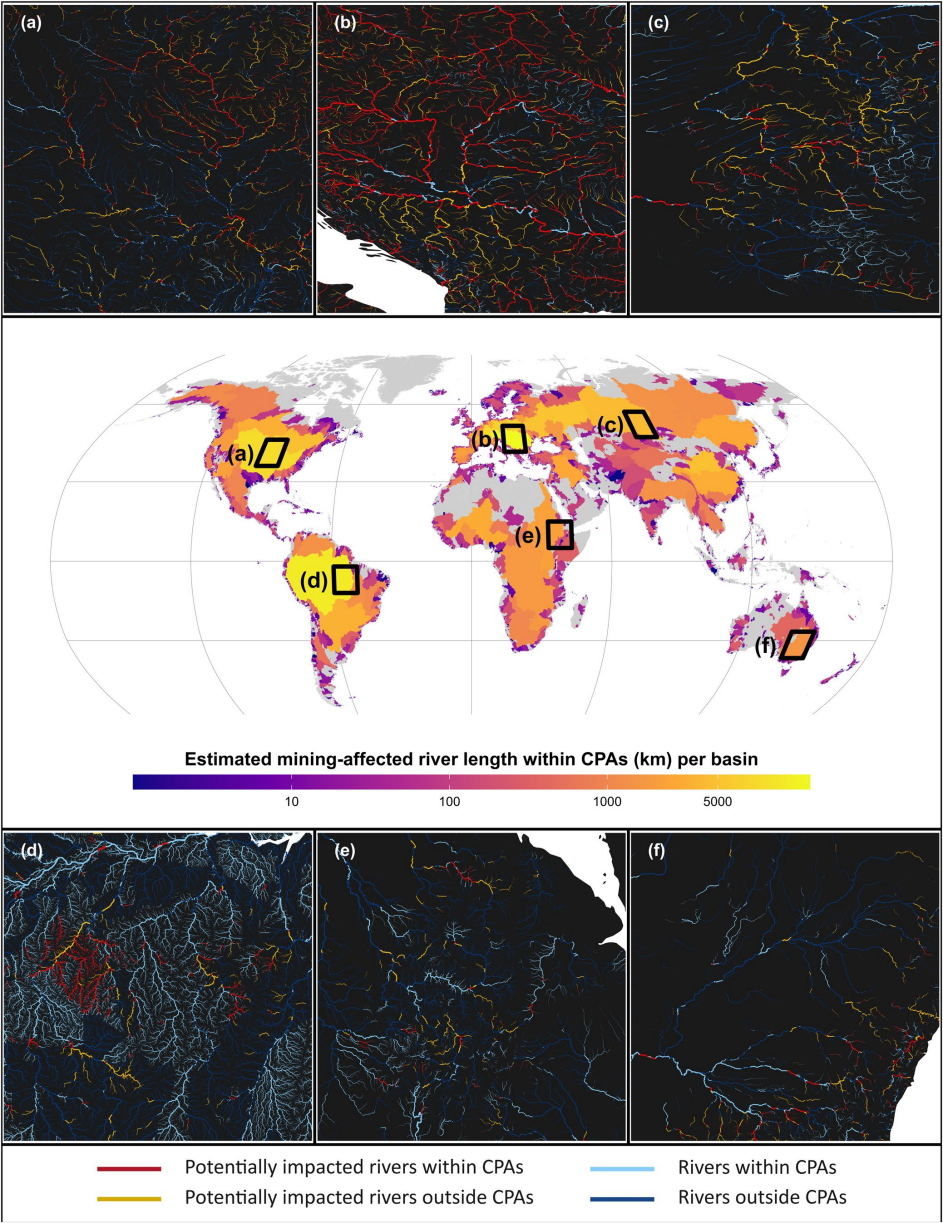
Distribution of rivers potentially exposed to mining‐related contamination within conservation priority areas (CPAs; i.e., the combined extent of Protected Areas and Key Biodiversity Areas), based on the severe modeling assumption. The middle panel presents a world map showing the total river length potentially exposed to mining impacts within CPAs, aggregated by hydrological basin. To enhance visualization, the scale is log_10_‐transformed, with labels expressed in kilometers for easier interpretation. The bounding boxes in the main map indicate the locations of the insets shown in the top (a–c) and bottom (d–f) panels. The insets provide detailed views of hydrological basins in each continent with a high estimated extent of overlap between potentially exposed river reaches and CPAs. In these zoomed‐in views, river line width is proportional to the annual average natural river discharge (Döll et al. 2003). For visualization purposes, only river segments with an annual average discharge greater than 0.5 m^3^/s are displayed.

The hydrological basins with the largest lengths of potentially exposed rivers overlapping with CPAs are concentrated in Central and Eastern Europe, with the Danube Basin ranking first (Figures 3 and S10). The Amazon Basin (South America) follows next, while the Mississippi Basin (North America) and the Yellow River Basin (Asia) are the only other non‐European basins in the top 10. Under the severe assumption, 33.5% of all river length within CPAs in the Danube Basin is potentially exposed to mining‐related contamination (19,000 km), compared to 1.3% in the Amazon Basin (14,000 km; Dataset S3). The primary commodities linked to this potential exposure differ between the two basins. In the Amazon, gold dominates, followed by copper and silver. In the Danube, gold is also prominent, alongside copper, nickel, cobalt, and coal. Additionally, overall mine sizes vary considerably: in the Amazon Basin, mines are, on average, larger (mean: ~65 km^2^, median: ~0.25 km^2^) compared to those in the Danube Basin (mean: ~0.18 km^2^, median: ~0.03 km^2^).

**FIGURE 4:**
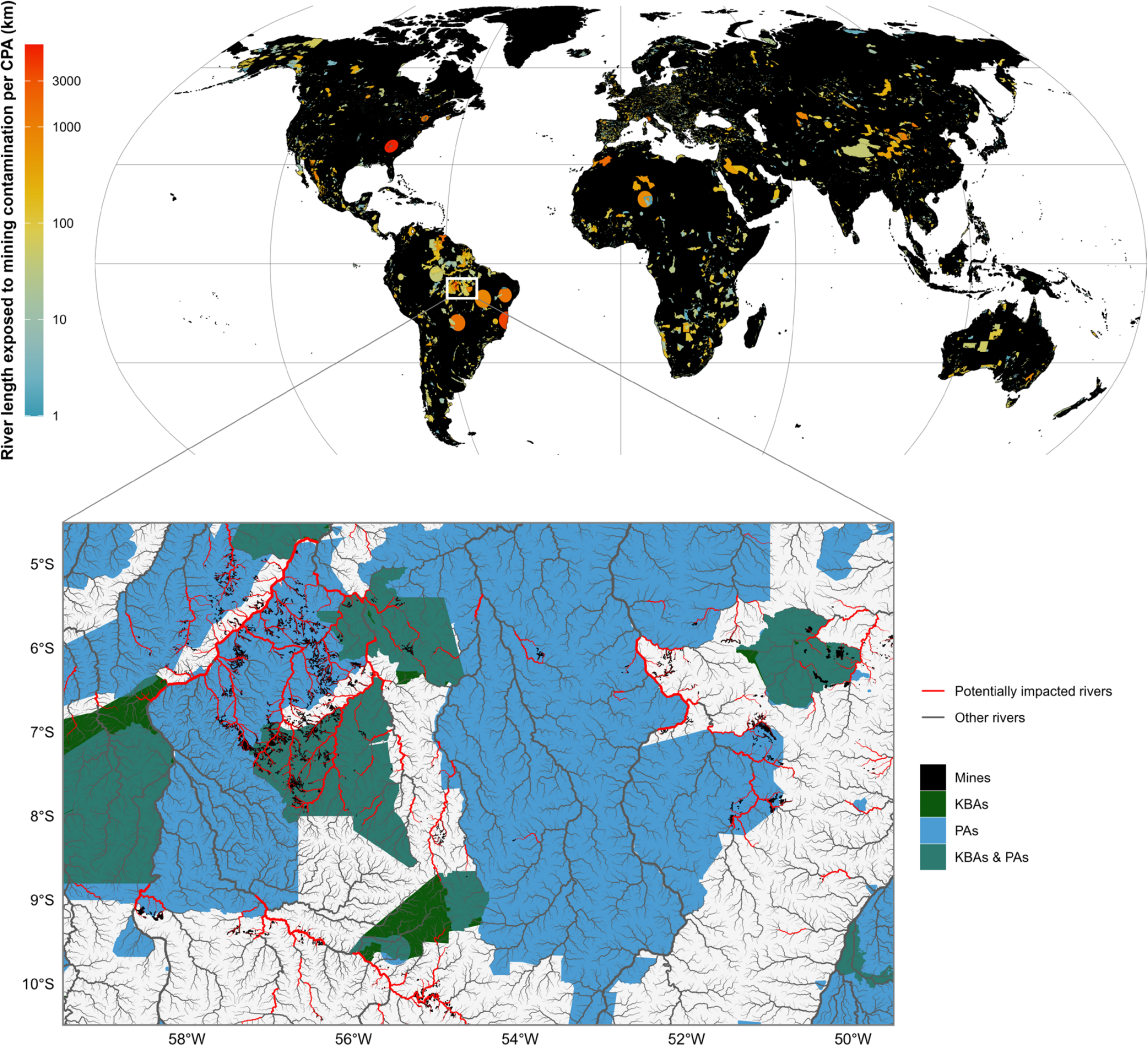
Global patterns of estimated river exposure to mining‐related contamination across conservation priority areas (CPAs). The main map shows CPAs, defined as the combined extent of Key Biodiversity Areas (KBAs) and Protected Areas (PAs), colored by the total length of rivers potentially exposed to mining contamination within each area. The color gradient represents log_10_‐transformed river length within each polygon under the severe modeling assumption, with values expressed in kilometers for interpretability. The inset highlights a region in Brazil containing CPAs with high projected exposure, and displays PAs, KBAs, and areas where they spatially overlap as separate categories. Black polygons indicate mine footprints, the majority of which are gold mines (primary commodity). For better visualization, only rivers with an annual average discharge greater than 0.5 m^3^/s are shown. Potentially exposed rivers are highlighted in red, while remaining rivers are shown in grey.

PAs and KBAs exhibit wide variability in both the absolute and relative extent of river networks potentially exposed to mining‐related contamination (Figures 4 and S11–S14; Datasets S4 and S5). Under the broader PA definition, the Southern Appalachian Biosphere Reserve (USA), a large UNESCO‐MAB designation without an assigned IUCN management category, records the highest extent of potentially exposed rivers, totaling approximately 6300 km (14.7% of its river network). Exposure within this site is dominated by coal mining and zinc to a lesser extent. The Tapajós Environmental Protection Area in Brazil ranks second under the broader PA definition. When restricting the analysis to IUCN‐categorized PAs, it is the most affected site, with approximately 4500 km (54.5%) of its river network potentially exposed. Gold mining is the predominant commodity affecting this PA, as well as several other highly exposed PAs in the Amazon region, including the Imataca Forest Reserve in Venezuela. The Novo Progresso KBA, which partially overlaps with the Tapajós Environmental Protection Area, shows the highest extent of potentially exposed rivers among KBAs, totaling over 2700 km (25.8%). Other highly exposed KBAs include Damaoqi in northern China, the Corredor de Barrancas de la Sierra Madre Occidental in Mexico, and the South‐west Slopes of New South Wales in Australia.

## 4 Discussion

This study provides a global‐scale assessment of areas of potential conflict between mining activities and freshwater biodiversity by identifying downstream rivers potentially affected by mining and evaluating their spatial overlap with CPAs (i.e., KBAs and PAs). It is important to emphasize that, although we distinguished among primary commodities to model the downstream extent of exposure, our model did not account for mining intensity or the duration of potential impacts. Therefore, our results should not be interpreted as reflecting the actual ecological impact of individual operations, given that impacts are expected to be strongest close to mining sites and to attenuate downstream as contaminant concentrations decline through dilution and sediment deposition. While we identified the extent of river systems potentially exposed to mining contamination, the magnitude of impact varies according to multiple factors, including mine characteristics, hydrological processes, species‐specific tolerances, and differences in environmental governance and regulatory enforcement (Ali et al. 2017; Butsic et al. 2015; Laurance 2004; Lin et al. 2022; Miranda et al. 2004; Sonter et al. 2018). Nonetheless, our findings serve as an important first step in identifying areas where freshwater biodiversity may be at risk, warranting further investigation at local, regional, and national scales, particularly through field‐based research.

Our analysis suggests a potential extent of downstream impacts approximately 2–3.7 times greater than the 480,700 km of river channels identified as affected by metal mining by Macklin et al. (2023). This difference is largely due to our inclusion of a more comprehensive mining dataset, which also accounts for non‐metal commodities such as coal and industrial minerals, as well as methodological differences in how potentially exposed rivers were identified. Additionally, our study addresses some of the data limitations of previous assessments, particularly in capturing the extent of small‐scale and artisanal mining, an issue raised in prior research (Macklin et al. 2023; Maus and Werner 2024; Murguía et al. 2016). This is especially important given that widely used industry datasets, such as S&P Global Market Intelligence (2024), are highly incomplete (Maus and Werner 2024). As a result, assessments based solely on these sources may overlook some of the most severely affected sites, particularly those associated with informal, illegal, abandoned, and underreported operations that often pose significant environmental risks.

A considerable fraction of river networks potentially exposed to mining‐related contamination in our analysis lies within CPAs (approximately 18%). When restricting the analysis to IUCN‐categorized PAs, which represent a more conservative and standardized definition of protection, significant overlap remains, with nearly 9% of potentially affected river length located within these areas. However, because many areas critical for freshwater biodiversity conservation extend beyond the standardized conservation designations represented by PAs and KBAs (Abell et al. 2008), and because the PA dataset still contains gaps in spatial coverage despite supplementation with national datasets, these values likely underestimate the overall extent of potential conflict between mining activities and freshwater biodiversity. Similar patterns have been reported for terrestrial ecosystems: Sonter et al. (2020) found that a notable share of land potentially influenced by mining coincides with PAs (8%) and KBAs (7%), while Murguía et al. (2016) reported that 23% of large‐scale metal mines and 20% of known ore deposits are located in areas of high terrestrial biodiversity. These findings suggest that while conservation areas may offer some degree of protection, they are not entirely exempt from mining‐related threats. In our analysis, this is evident even within PAs with assigned IUCN management categories, as illustrated by the Tapajós Environmental Protection Area in Brazil. Within this PA, thousands of kilometers of rivers are potentially exposed to mining‐related contamination, accounting for nearly half of its total river network. Evidence of enduring mercury contamination and physiological damage in fish has been reported in the Tapajós Basin decades after artisanal mining was officially prohibited (da Silva Montes et al. 2022), exemplifying how mining‐related impacts can persist long after formal restrictions are imposed.

These patterns underline fundamental shortcomings in conservation strategies for freshwater ecosystems. Despite international commitments such as the Kunming‐Montreal Global Biodiversity Framework's target to protect 30% of inland waters by 2030, current coverage remains below 15%, with implementation lagging behind policy ambitions (CBD 2022). Yet beyond this shortfall in coverage, our findings also question the effectiveness of areas that are already designated as protected. Although PAs benefit from legal designation, enforcement is frequently limited, particularly with regard to freshwater systems (Acreman et al. 2020). These limitations help explain why informal artisanal and small‐scale mining is frequently observed within these areas, facilitated by inadequate monitoring, displacement from industrial concessions, and perceptions that PAs are more accessible or loosely regulated than other lands (Dossou Etui et al. 2024). At the same time, conservation planning tends to prioritize terrestrial habitats and charismatic species, resulting in systematic underrepresentation of freshwater ecosystems (Darwall et al. 2011; Delso et al. 2021; Nogueira et al. 2021). This limited coverage is especially concerning given the long‐distance transport of pollutants through river networks (Bernhardt and Palmer 2011), which can expose protected freshwater systems to mining impacts far downstream. As such, while expanding the global network of PAs is necessary, it must be guided by considerations of hydrological connectivity and the downstream transport of pollutants. Equally important is the implementation of measures to ensure that designated areas are effective in mitigating mining‐related impacts.

Our results indicate that among the subset of mines with identified primary commodities, gold mining is associated with the most extensive exposure of river systems to potential contamination, both globally (approximately 275,000 km under the severe assumption) and within CPAs (46,000 km). However, these estimates likely underestimate the full extent of potential gold mining‐related contamination, as a large fraction of the mines in our dataset lack primary commodity information. These sites likely include quarrying operations, legacy mines, and small‐scale or informal activities, among which unregistered gold mining may play an important role (see SI Extended Methods for further details). This is particularly plausible in countries like Brazil and Peru, where many of the unknown‐commodity mines are located and where informal gold mining is prevalent and often unmonitored.

Gold extraction poses serious environmental risks, particularly through the use of mercury and cyanide in ore processing, which contribute to long‐term contamination of aquatic ecosystems and risks to biodiversity and human health (Dossou Etui et al. 2024). While large‐scale gold mining is often regulated, its environmental impact remains significant, especially in regions where compliance and enforcement challenges persist (Murguía et al. 2016; Usman Kaku et al. 2021). Although a lower proportion of gold deposits are in high‐biodiversity zones compared to the proportion of operating gold mines, large‐scale mining still overlaps with ecologically sensitive areas, and its presence may facilitate further expansion of artisanal and small‐scale gold mining (Murguía et al. 2016). These small‐scale mining operations have expanded rapidly in recent decades, often within PAs and biodiversity hotspots (Alvarez‐Berríos and Mitchell Aide 2015; Asner and Tupayachi 2016; Dossou Etui et al. 2024). The widespread and predominantly unregulated nature of small‐scale gold mining raises concerns about cumulative environmental impacts, particularly mercury contamination in freshwater systems and increased sedimentation in riverine habitats (Lobo et al. 2019; Markham and Sangermano 2018; Martinez et al. 2018; Ngom et al. 2023). Governance challenges and conflicts with large‐scale mining companies further drive artisanal and small‐scale gold mining expansion into ecologically sensitive areas (Dossou Etui et al. 2024). Although illegal gold mining can be more environmentally disruptive than large‐scale operations, it is often excluded from large‐scale assessments due to data limitations. However, our dataset captures at least part of these activities, identifying areas of potential conflict between gold extraction and freshwater biodiversity that warrant further investigation. Despite its significant environmental footprint, gold plays a negligible role in the global energy transition, as it is not required for decarbonization technologies and has limited industrial applications (Trench et al. 2024). Its value is predominantly financial and symbolic rather than functional, which underscores the need to critically evaluate the environmental costs of gold production in relation to its minimal contribution to sustainable development objectives.

Although the global shift toward clean energy aims to reduce coal mining in the long term (IEA 2024), its influence on freshwater ecosystems remains extensive, ranking second only to gold in our analysis. An estimated 165,000 km of river networks may be at risk of contamination from coal mining activities, including nearly 22,000 km within CPAs. Similarly, key Energy Transition Mineral (ETM) mining may expose an estimated 142,000 km of rivers to contamination, with 23,000 km within CPAs. This suggests that, despite growing attention on the environmental costs of key ETM extraction, coal mining alone potentially remains a dominant driver of freshwater contamination. In addition, while the estimated extent of rivers potentially affected by key ETM mining is comparable to that of coal, only up to 25% can be attributed to the current demand for clean energy technologies. Although this proportion is likely an overestimate, as our dataset does not account for whether the extracted minerals are in forms suitable for energy applications, it highlights that, currently, most ETM‐related mining activity and its associated impacts are driven by demands beyond clean energy technologies.

Both coal and key ETM mining have been associated with the degradation of freshwater systems, namely through acid mine drainage, heavy metal contamination, sedimentation, and hydrological disruptions, posing serious threats to biodiversity (Affandi and Ishak 2019; Giam et al. 2018; de Haes and Lucas 2024; IEA 2024; Simonin et al. 2021; Sonter et al. 2020). As future demand for key ETMs is expected to rise substantially (IEA 2023), these minerals are likely to play an increasingly dominant role in global ecological impacts, making it crucial to investigate the potential effects of future mining on biodiversity. At the same time, key ETMs are characterized by high price volatility and rapidly changing market conditions, which can drive shifts in production levels, operational decisions, and ownership structures, potentially influencing the intensity of pressures on freshwater ecosystems. Although the energy transition is essential, it must be accompanied by biodiversity‐inclusive strategies to minimize ecosystem harm. Strategic mine site selection, improved environmental regulations, increased transparency, downstream environmental monitoring, and conservation planning are crucial to reducing conflicts with freshwater ecosystems and mitigating biodiversity loss. To better inform these strategies, further research is needed to link market dynamics to freshwater ecological impacts. Moreover, engaging mining companies, policymakers, and conservation organizations in proactive discussions is paramount to reconcile mineral demands with biodiversity conservation (Sonter et al. 2018).

Ensuring that conservation measures and regulatory frameworks effectively mitigate mining‐related threats requires not only addressing active operations but also understanding the extent and persistence of impacts from inactive mines. Our ability to fully assess these impacts is constrained by data limitations, as a large proportion of mines in our dataset lack classification regarding their activity status. Despite this uncertainty, among the mines we were able to classify, our findings suggest that inactive mines may contribute more extensively to potentially affected river lengths than active mines, in line with previous research (Macklin et al. 2023). The persistence of mining‐related contamination long after operations cease has been well documented, with studies showing that both chemical and sediment pollution can continue to affect freshwater systems for decades, particularly where remediation efforts are inadequate (Atibu et al. 2016; Bao et al. 2023; Bennett 2016; Price and Wright 2016; Wright and Burgin 2009). In some cases, even reclaimed mines (e.g., coal mines) continue to show reduced stream biodiversity compared to unmined systems, reflecting concerns that current regulatory frameworks may be insufficient to fully mitigate long‐term mining impacts (Giam et al. 2018). This underscores the need for long‐term ecological assessments, as the effectiveness of post‐mining restoration remains poorly understood. Yet, long‐term monitoring of mine restoration is scarce, making it difficult to determine whether rehabilitated ecosystems can truly sustain freshwater biodiversity (Harries et al. 2024). Restoration assessments often emphasize land cover recovery while overlooking other key attributes such as ecosystem functioning and species composition, limiting our ability to evaluate post‐mining ecosystem resilience (Cristescu et al. 2012; McKenna et al. 2020). At the same time, regulatory frameworks for mine closure are often inconsistent, with financial assurances frequently insufficient to cover long‐term rehabilitation costs, leading to ongoing pollution risks from inactive mines (Young et al. 2022). Despite these challenges, our study provides a spatial perspective by pinpointing areas of high conservation value where mining‐related contamination may persist, highlighting priority regions for further research and conservation efforts. Strengthening mine closure regulations, enforcing long‐term remediation strategies, and integrating legacy mining impacts into biodiversity conservation efforts will be critical to mitigating freshwater contamination and ecosystem degradation.

Beyond remediation and regulatory reform, strategies to reduce primary materials demand and therefore the overall scale of mining remain a critical component in limiting environmental impacts. Shifting toward more resource‐efficient systems, such as smaller and lighter vehicles, expanded public transport networks, and improvements in energy efficiency, can significantly reduce the need for mineral extraction (Riofrancos et al. 2023). Technology choices also influence mining pressures. For instance, lithium iron phosphate (LFP) batteries do not require cobalt, nickel, or manganese, and their wider adoption could reduce reliance on some of the most environmentally damaging supply chains (Llamas‐Orozco et al. 2023). In parallel, circular economy approaches that prioritize mineral reuse, repurposing, remanufacturing, and recycling offer important opportunities to reduce pressure on ecosystems (Sovacool et al. 2020). Incorporating these upstream strategies into energy transition planning is essential to minimizing future mining expansion and aligning climate goals with freshwater biodiversity protection.

## Author Contributions

M.B.P.: conceptualization, formal analysis, investigation, methodology, software, validation, visualization, writing – original draft, writing – review and editing. N.B.: conceptualization, methodology, writing – review and editing. A.M.: supervision, writing – review and editing. R.K.: supervision, writing – review and editing. M.B.: supervision, writing – review and editing. V.M.: Data curation, writing – review and editing. V.B.: conceptualization, methodology, supervision, writing – review and editing.

## Funding

This work was supported by Nederlandse Organisatie voor Wetenschappelijk Onderzoek, 19920 VI.Veni.232.094.

## Conflicts of Interest

The authors declare no conflicts of interest.

## Supporting information


**Appendix S1:** gcb70774‐sup‐0001‐AppendixS1.pdf.

## Data Availability

The data and code that support the findings of this study are available in the Zenodo repository at: https://doi.org/10.5281/zenodo.18659366.
