# Supplementary material for: Freshwater Conservation Priority Areas Are Threatened by Global Mining Activities
Source: Glob Chang Biol. 2026 Mar 3;32(3):e70774. doi: 10.1111/gcb.70774 (PMC12954647; doi:10.1111/gcb.70774)
Supplement: Supplementary file 1 — Appendix S1: gcb70774‐sup‐0001‐AppendixS1.pdf. [file GCB-32-e70774-s001.pdf]

Supplementary Information

Freshwater conservation priority areas are threatened by global mining activities

Mariana Braz Pires[a,*], Nina Baltus[a], Alexandra Marques[b], Rene Kleijn[a], Mike Buxton[c], Victor Maus[d,e], Valerio Barbarossa[a,b]

[a] Institute of Environmental Sciences, Leiden University, P.O. Box 9518, 2300 RA Leiden, The Netherlands
[b] Global Sustainability, PBL Netherlands Environmental Assessment Agency, The Hague, The Netherlands
[c] Geoscience and Engineering Department, Delft University of Technology, Stevinweg 1, 2628 CN Delft, The Netherlands
[d] Institute for Ecological Economics, Vienna University of Economics and Business, Vienna 1020, Austria
[e] Novel Data Ecosystems for Sustainability Group, Advancing Systems Analysis, International Institute for Applied Systems Analysis, Laxenburg 2361, Austria

* Corresponding author
E-mail address: m.braz.pires@cml.leidenuniv.nl

Extended methods

Mines with unknown commodities

In the global georeferenced mining dataset used in our study (*1*), more than half of all mine polygons lack an associated commodity, although they represent only 22.5% of the total mapped mining area. This reflects a broader issue in global mining data: recent research has shown that 56% of the world's mapped mining areas have no associated commodity or production data in global compilations such as S&P database (*2*), with missing information distributed across all regions and commodity types (*3*). Based on available evidence, we expect that many of the unlabeled polygons correspond to small-scale or informal operations, quarrying, or legacy sites. Although it is possible that some large-scale industrial mines are not captured in global databases, most such operations, particularly those producing internationally traded metals like copper or iron, are generally reported. The following brief exploration provides an overview of the characteristics and spatial distribution of the unknown group (i.e., the group of mines without known commodities in our dataset) to contextualize their likely origin.

To better understand the nature of these unlabeled features, we compared the area of mines with and without reported commodities (Table S3). On average, unknown-commodity polygons are considerably smaller (median: 3.6 hectares versus 6.1 hectares; mean: 30.1 hectares versus 111.7 hectares). To quantify the uncertainty around these differences, we applied a bootstrapping approach by drawing 1,000 random samples from each group and repeating the process 10,000 times to estimate confidence intervals for the mean and median values. Bootstrapping was selected over standard hypothesis tests because, given the very large sample sizes (over 100,000 observations per group), even minimal differences may result in extremely small p-values, potentially overstating their practical importance. The bootstrapped estimates yielded 95% confidence intervals of [64.1, 214.8] hectares for the mean area in the known group and [17.1, 67.4] hectares for the unknown group. For medians, the intervals were [5.1, 7.12] and [3.1, 4.2] hectares, respectively, with a median difference of 2.44. These results confirm a systematic, though modest, difference in area between the two groups, with the unknown-commodity polygons skewing toward smaller scale operations.

To explore spatial patterns, we examined the distribution of unknown-commodity polygons by country. The countries that account for the largest total area of such polygons are Russia, the United States, Peru, China, Myanmar, and Brazil, together containing 51.7% of the global extent of unknown-commodity mining areas (Tables S4-S5). Across these contexts, the absence of commodity information is likely explained by a combination of widespread quarrying for construction materials, informal or artisanal and small-scale mining that escapes official reporting, and legacy or inactive operations lacking modern records. In Peru and Brazil, where illegal and small-scale gold mining is known to be widespread across parts of the Amazon region, many mining operations remain unregistered and unmonitored, making it plausible that a portion of the unknown-commodity polygons in these countries corresponds to this underreported sector (*4*–*7*). In Myanmar and Russia, the high proportion of unknown-commodity polygons likely reflects the presence of informal mining and limited transparency, including the lack of accessible mine registries and reporting frameworks (*8*–*10*). In China and the United States, many of the mine polygons without reported commodities likely reflect a mix of quarrying for construction materials, small-scale extraction, and legacy or abandoned sites, all of which are poorly captured in global databases (*11*–*13*).

Randomization: mine-to-river linkage

To evaluate the robustness of the method used to link mine polygons to downstream river segments, we conducted a randomization-based validation analysis. This analysis aimed to determine whether the strategy applied in the main analysis, which combines centroid-based and intersection-based approaches, reliably identifies the most probable hydrological connections between mining areas and rivers.

The validation procedure was implemented by first rasterizing all mine polygons at a 15 arc-second resolution, consistent with the resolution of the flow direction data used throughout the study. Within each mine polygon, we randomly sampled up to 999 points, ensuring that only one point was drawn from each raster cell. This constraint prevented artificial duplication of discharge locations from within the same cell, which would otherwise bias the results by repeatedly routing identical or nearly identical entry points to the same river segment. For smaller mines containing fewer than 999 raster cells, a single point was drawn from each cell without repetition, thereby maintaining spatial diversity while avoiding redundancy across the sampled points. Each sampled point was then treated as a potential discharge location and routed downslope using the same hydrological model and flow direction data applied in the main analysis.

We evaluated the similarity between the main linkage method and the randomized validation approach by calculating four standard metrics. Recall, defined as the proportion of river reaches identified by the main method that also appeared in the randomized set, was 98.5%. This indicates that nearly all segments captured by the linkage approach were also recovered through random sampling. Precision, defined as the proportion of river segments identified through randomization that were also found by the main method, was 79.3%. Together, these two values reflect a high degree of coverage and specificity. The harmonic mean of precision and recall (the F1 score) was 87.8%. The Jaccard similarity index, which measures the size of the intersection divided by the size of the union of the two sets of river segments, was 0.783.

Together, these values demonstrate that the mine-to-river linkage method used in the main analysis provides a robust and efficient representation of downstream connectivity. The high recall indicates that very few valid linkages were missed, while the relatively high precision confirms that the approach did not over-identify river segments compared to the more spatially flexible random sampling procedure. These results support the use of the centroid- and intersection-based linkage strategy as a reliable and computationally efficient method for large-scale hydrological exposure modeling.

SI Figures

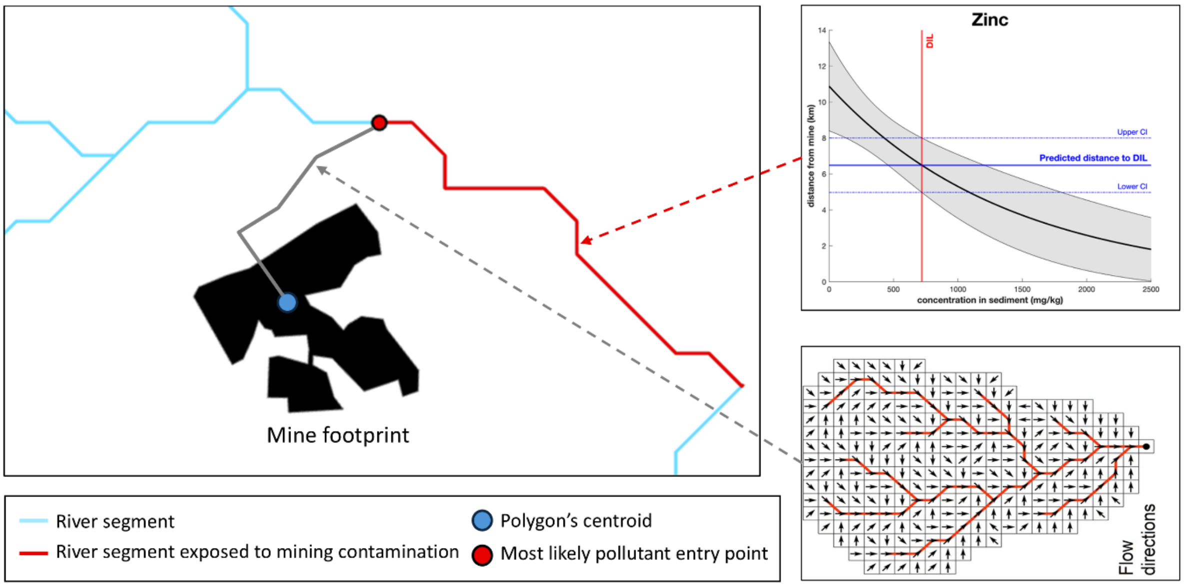

Figure S1. *Schematic overview of the spatial modelling framework used to estimate potential downstream exposure of river segments to mining-related contamination. The left panel shows a mine footprint (black polygon), its centroid (blue point), the most likely pollutant entry point on the river network (red point), and the downstream river segments potentially exposed to mining contamination (red lines). The grey path between the centroid and the river illustrates the downslope routing derived from the flow-direction grid (lower-right panel) based on HydroATLAS data (14). The upper-right panel presents an example attenuation curve for zinc retrieved from Macklin et al. (15), illustrating how predicted distances to the Dutch Intervention Limit (DIL) were used to derive commodity-specific attenuation thresholds.*

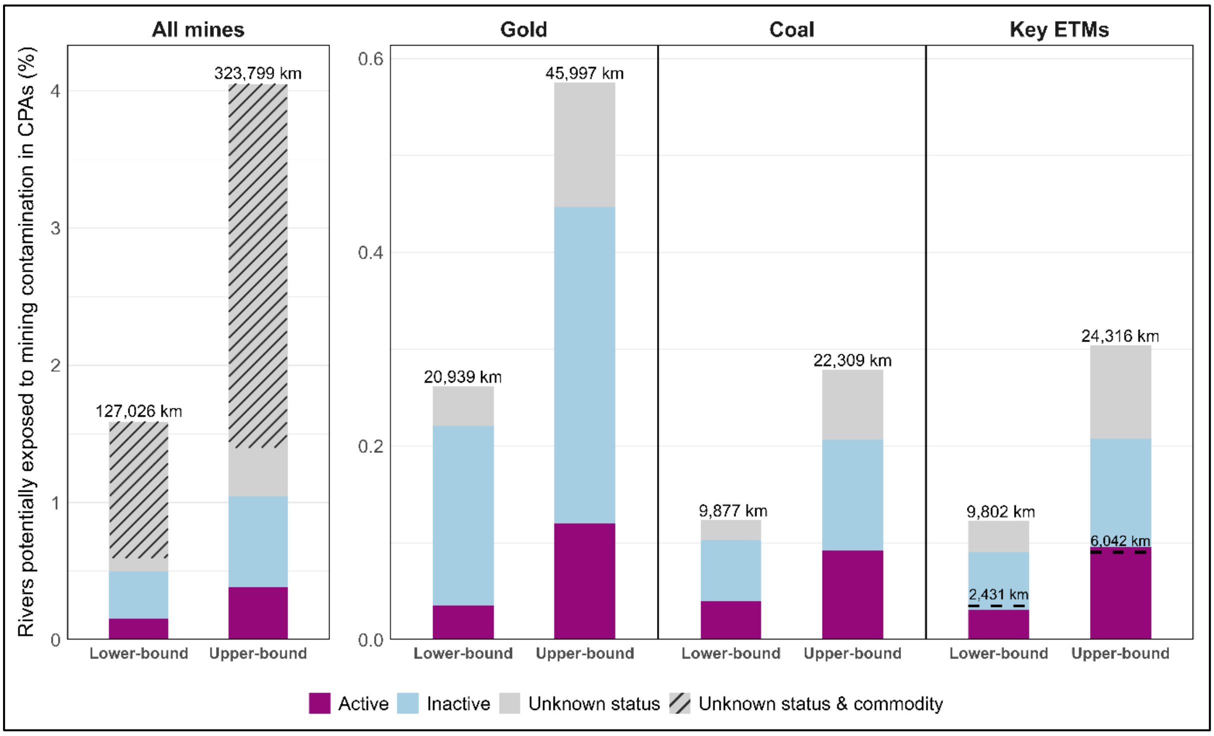

Figure S2. *Bar charts showing the proportion of global river length within conservation priority areas (CPAs) potentially exposed to mining-related contamination. CPAs are defined as the combined extent of Protected Areas and Key Biodiversity Areas. The figure includes estimates under lower-bound and upper-bound assumptions. Results are presented for all mines (left panel) and for three subsets of mines (right panels): coal, gold, and key energy transition mineral (ETM) mines. Bar heights represent the percentage of river length within CPAs that is potentially impacted by mining activities, calculated as the ratio of potentially exposed river length within these areas to the total river length across all CPAs. Each bar separates the contribution of active, inactive, and mines with unknown activity status, including mines with unknown primary commodities (a subset of mines with unknown activity status). Absolute lengths of rivers potentially exposed to downstream mining contamination (km) are displayed on top of each bar. In the chart focused on key ETM mines, the area below the dashed black line indicates the estimated river length potentially exposed to contamination from mining of minerals used in clean energy technologies, based on 2023 demand estimates from the IEA (16). This portion includes all activity statuses, while the remaining bar segment reflects additional potential exposure from mining of these minerals for other uses.*

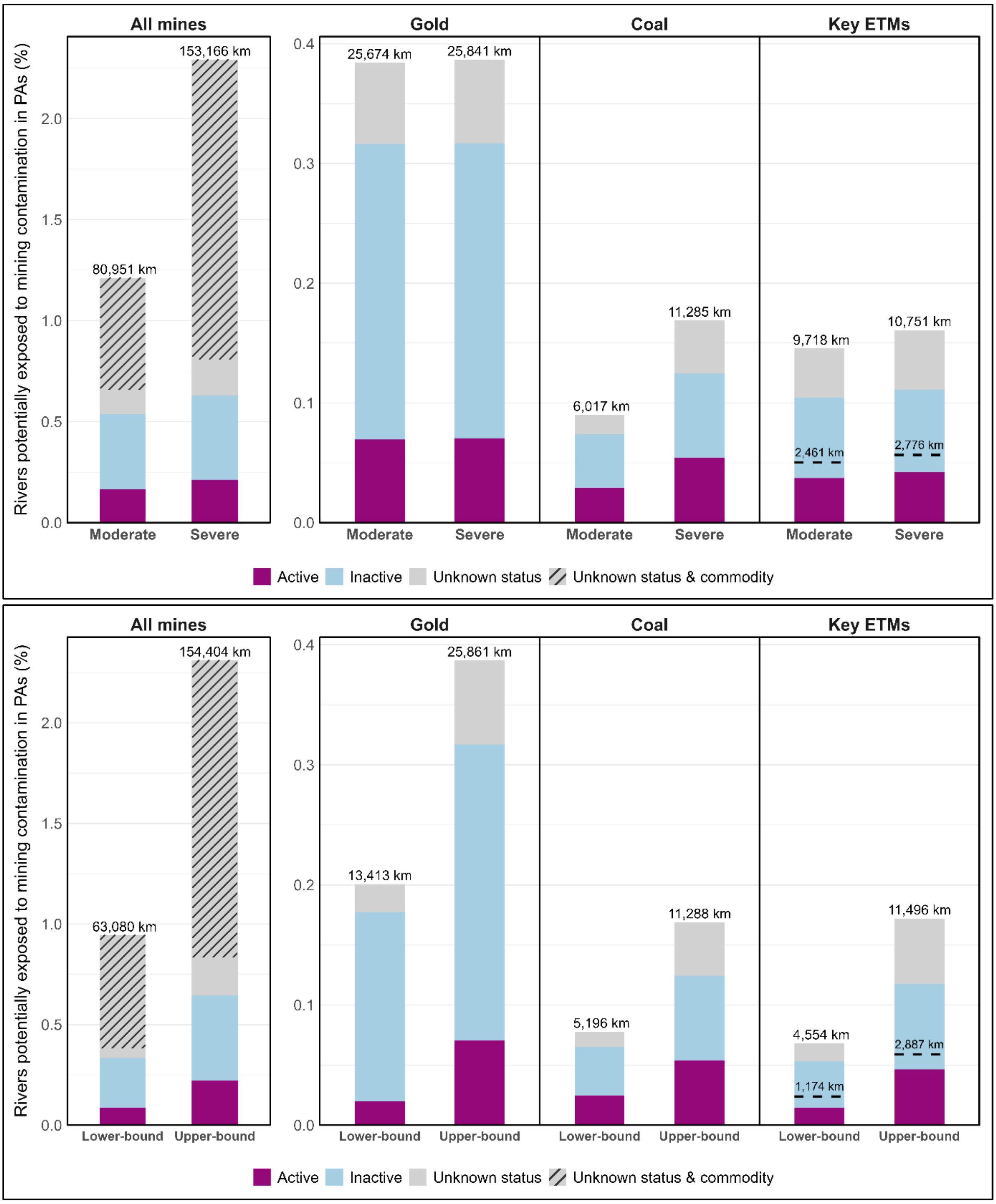

Figure S3. *Bar charts showing the proportion of global river length IUCN-categorized protected areas potentially exposed to mining-related contamination. The top panel shows results under the moderate and severe downstream contamination modeling assumptions, presented for all mines (left) and for three subsets of mines (right): coal, gold, and key energy transition mineral (ETM) mines. The bottom panel shows results under the lower-bound and upper-bound modeling assumptions for the same mine groups. Bar heights represent the percentage of river length within protected areas that is potentially impacted by mining activities, calculated as the ratio of potentially exposed river length within these areas to the total river length across all protected areas. Bars separate the contributions of active, inactive, and mines with unknown activity status, including mines with unknown primary commodities (a subset of mines with unknown activity status). Absolute lengths of rivers potentially exposed to downstream mining contamination (km) are displayed on top of each bar. In the charts focused on key ETM mines, the area below the dashed black line indicates the estimated river length potentially exposed to contamination from mining of minerals used in clean energy technologies, based on 2023 demand estimates from the IEA (16). This portion includes all activity statuses, while the remaining bar segment reflects additional potential exposure from mining of these minerals for other uses.*

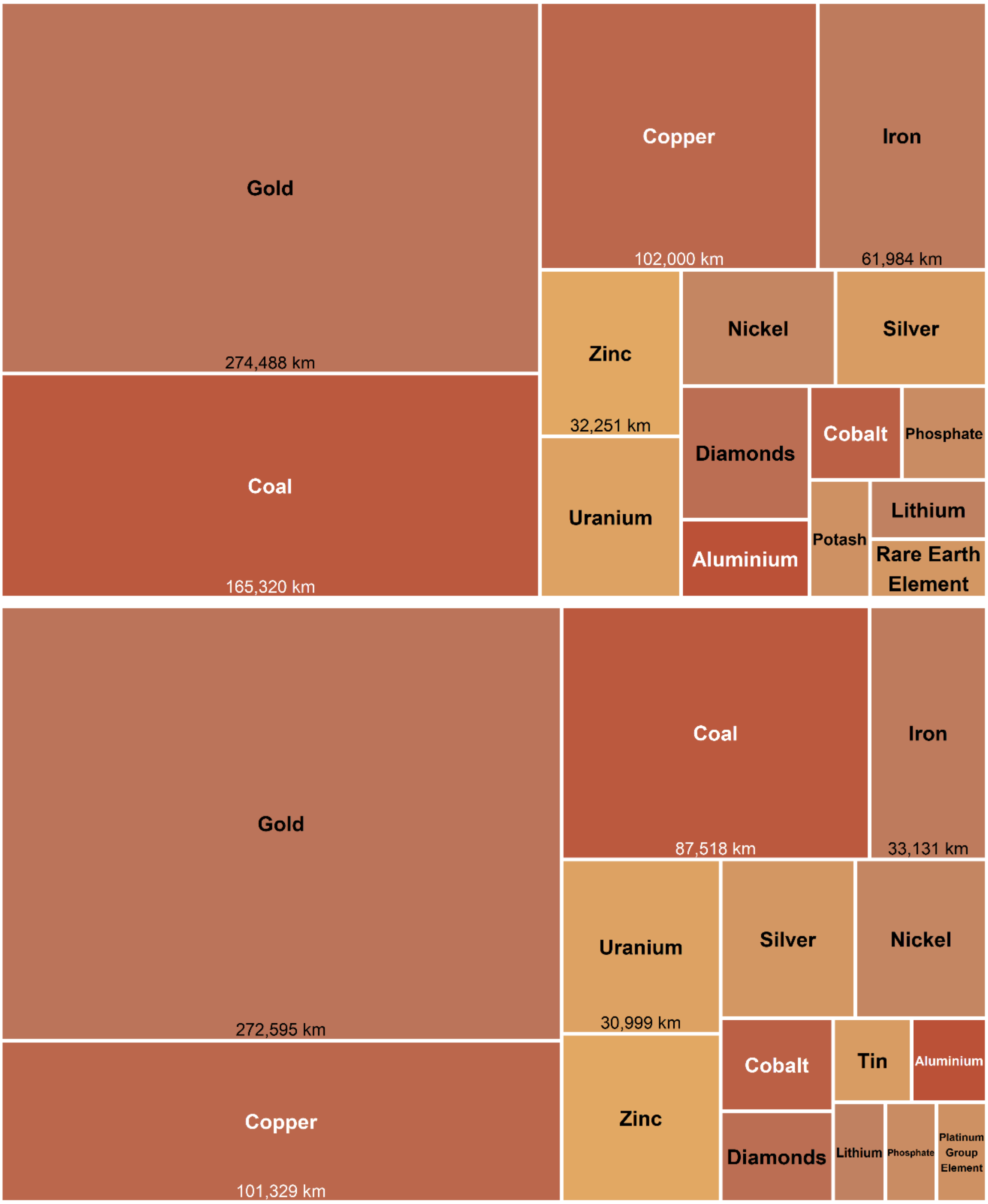

Figure S4*. Relative contribution of primary mining commodities to the estimated global river length potentially exposed to mining-related contamination. The top panel shows results based on the severe modeling assumption, and the bottom panel based on the moderate modeling assumption. Each panel displays the top 15 primary mining commodities with the highest estimated contribution under each assumption. Box sizes represent each commodity's share of the total potentially contaminated river length. Because multiple mining commodities may influence a single river segment, values are not mutually exclusive. To improve clarity, the figure excludes the Unknown category (representing 1,256,869 km in the top panel and 485,220 km in the bottom panel), where data on the primary commodity of the mine were unavailable.*

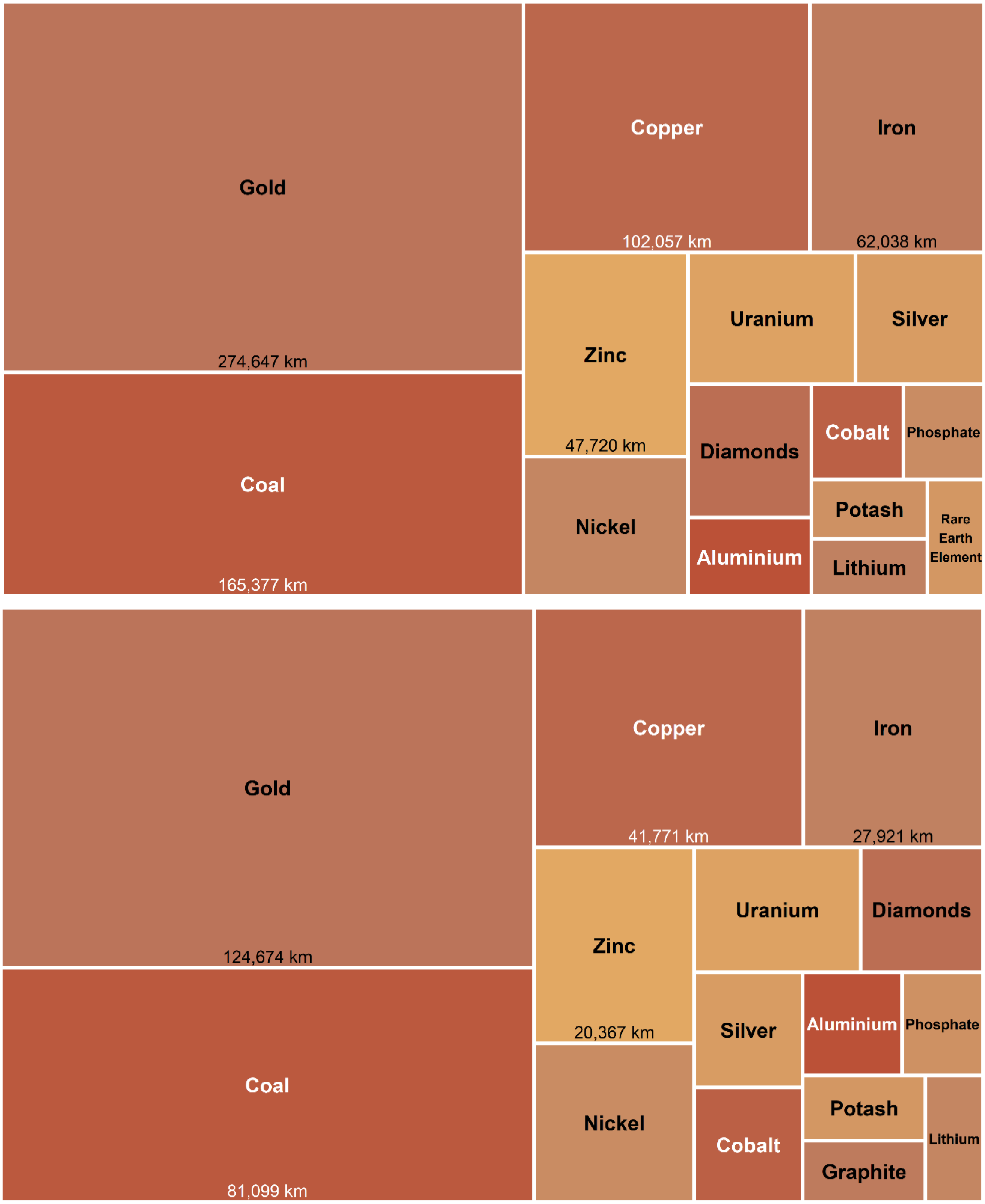

Figure S5. *Relative contribution of primary mining commodities to the estimated global river length potentially exposed to mining-related contamination. The top panel shows results based on the upper-bound modeling assumption, and the bottom panel based on the lower-bound modeling assumption. Each panel displays the top 15 primary mining commodities with the highest estimated contribution under each assumption. Box sizes represent each commodity's share of the total potentially contaminated river length. Because multiple mining commodities may influence a single river segment, values are not mutually exclusive. To improve clarity, the figure excludes the Unknown category (representing 1,259,596 km in the top panel and 440,317 km in the bottom panel), where data on the primary commodity of the mine were unavailable.*

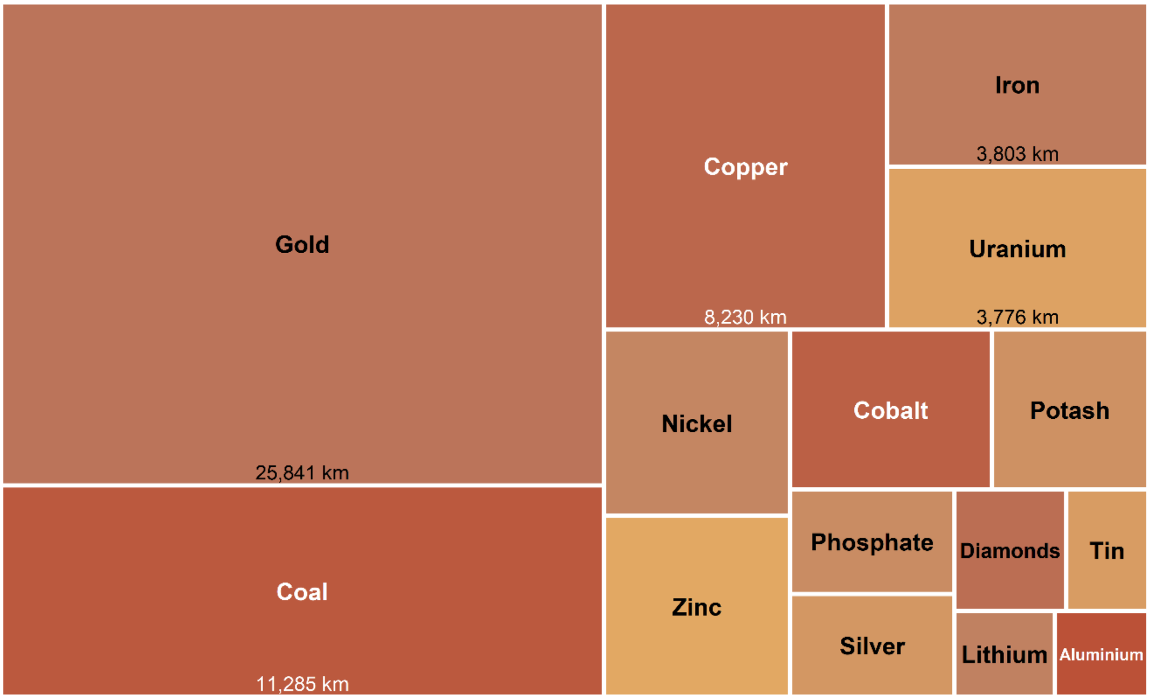

Figure S6. *Relative contribution of primary mining commodities to the estimated river length potentially exposed to mining-related contamination within IUCN-categorized protected areas, based on the severe modeling assumption. The figure displays the top 15 primary mining commodities with the highest estimated contribution. Box sizes represent each commodity's share of the total potentially contaminated river length within protected areas. Because multiple mining commodities may influence a single river segment, values are not mutually exclusive. To improve clarity, the figure excludes the Unknown category (representing 115,730 km of river length potentially influenced by mining), where data on the primary commodity of the mine were unavailable.*

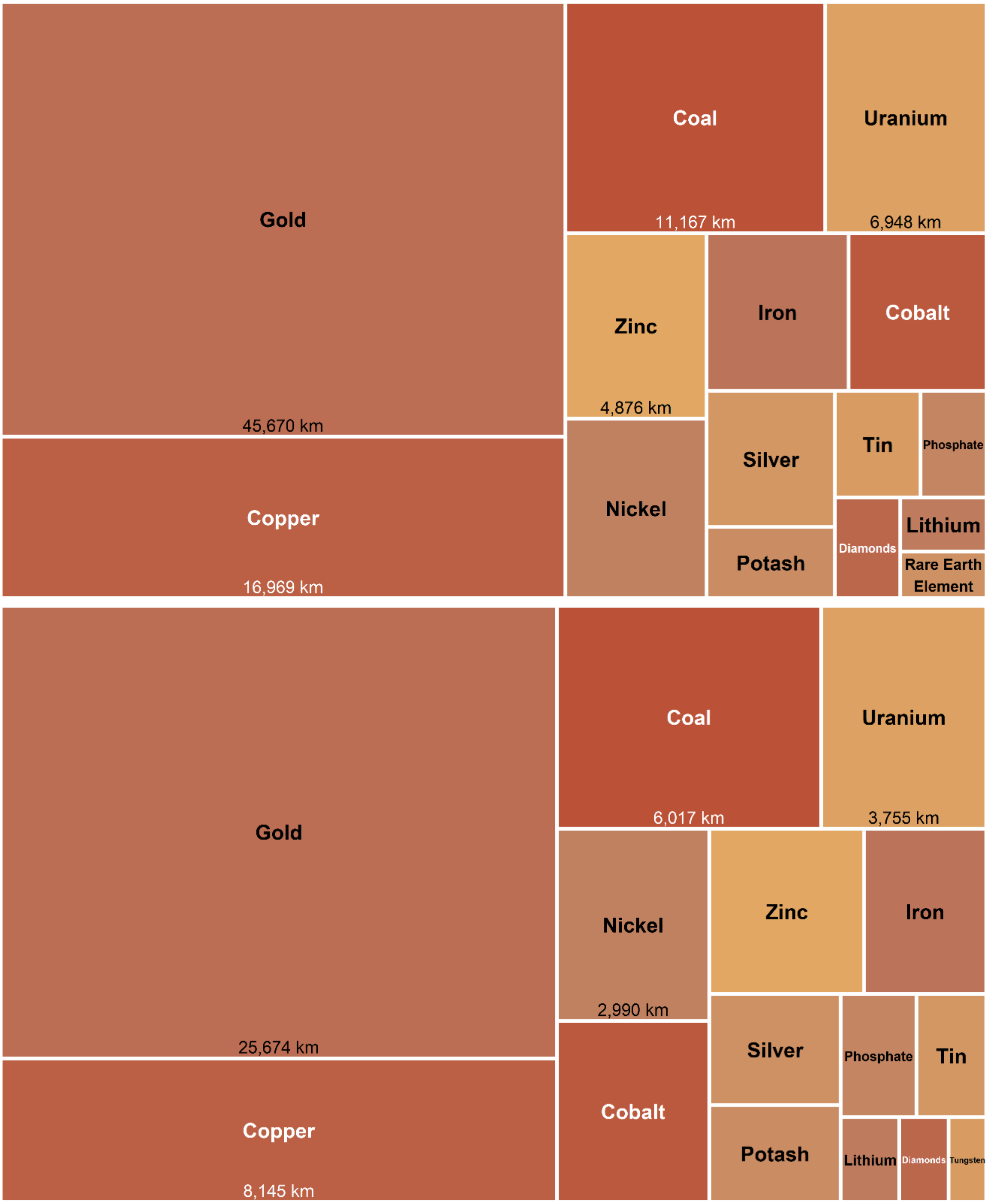

Figure S7*. Relative contribution of primary mining commodities to the estimated river length potentially exposed to mining-related contamination, based on the moderate modeling assumption. The top panel shows results for river segments located within conservation priority areas, defined as the combined extent of protected areas and key biodiversity areas. The bottom panel shows results for river segments located within IUCN-categorized protected areas. The figure displays the top 15 primary mining commodities with the highest estimated contribution in each panel. Box sizes represent each commodity's share of the total potentially contaminated river length in the respective spatial context. Because multiple mining commodities may influence a single river segment, values are not mutually exclusive. To improve clarity, the figure excludes the Unknown category (representing 897,878 km in the top panel and 44,283 km in the bottom panel), where data on the primary commodity of the mine were unavailable.*

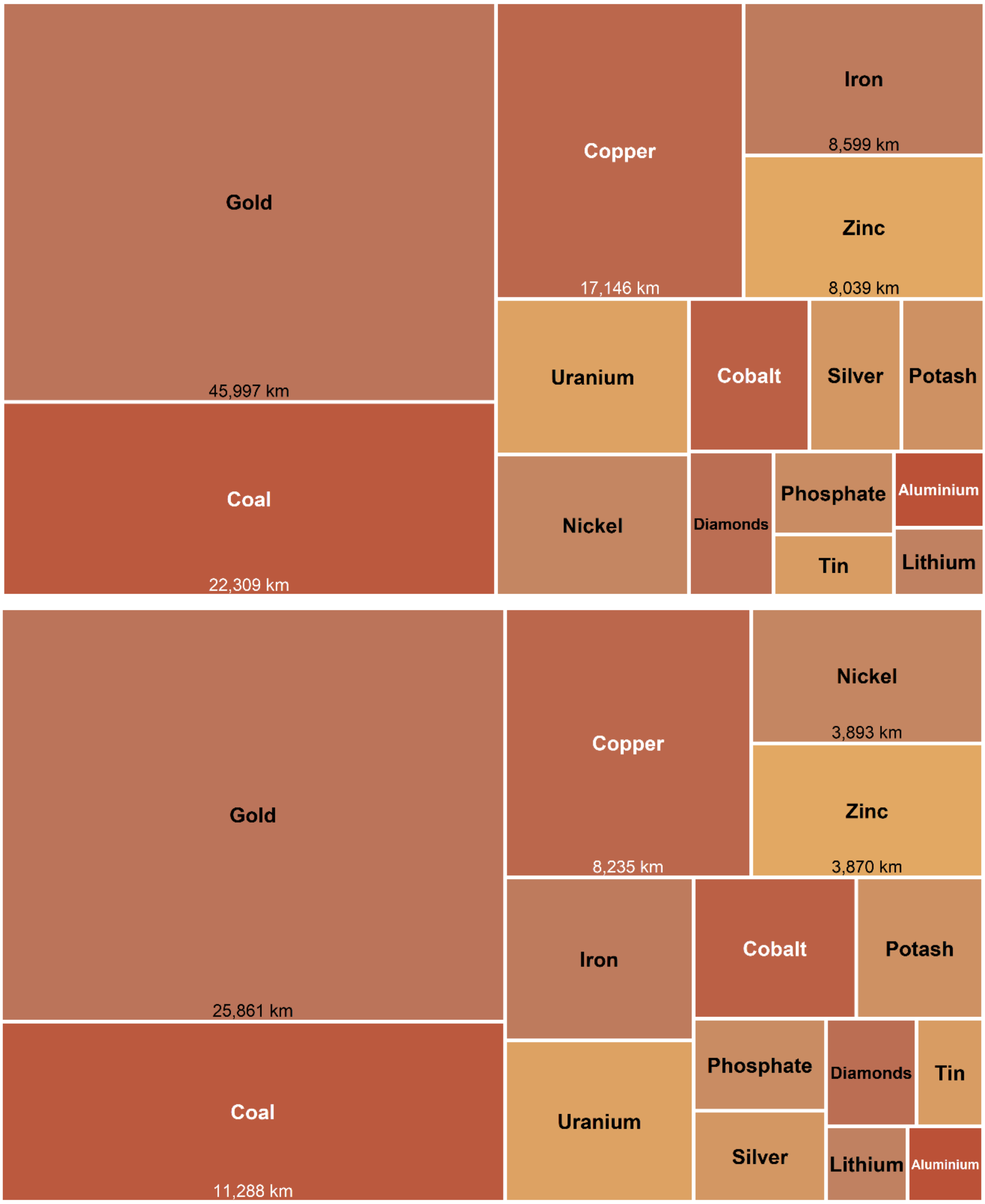

Figure S8. *Relative contribution of primary mining commodities to the estimated river length potentially exposed to mining-related contamination, based on the upper-bound modeling assumption. The top panel shows results for river segments located within conservation priority areas, defined as the combined extent of protected areas and key biodiversity areas. The bottom panel shows results for river segments located within IUCN-categorized protected areas. The figure displays the top 15 primary mining commodities with the highest estimated contribution in each panel. Box sizes represent each commodity's share of the total potentially contaminated river length in the respective spatial context. Because multiple mining commodities may influence a single river segment, values are not mutually exclusive. To improve clarity, the figure excludes the Unknown category (representing 243,811km in the top panel and 115,920 km in the bottom panel), where data on the primary commodity of the mine were unavailable.*

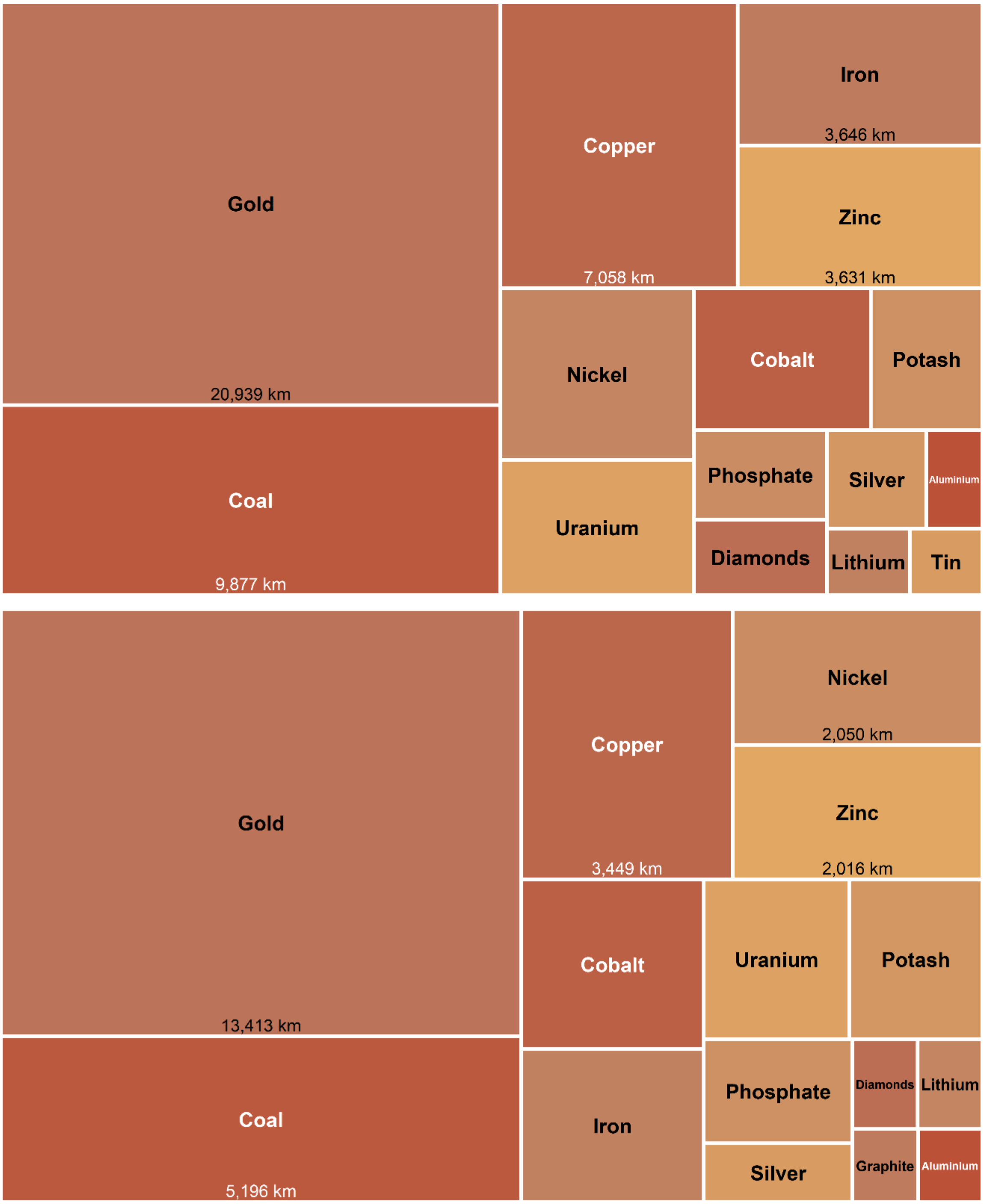

Figure S9. *Relative contribution of primary mining commodities to the estimated river length potentially exposed to mining-related contamination, based on the lower-bound modeling assumption. The top panel shows results for river segments located within conservation priority areas, defined as the combined extent of protected areas and key biodiversity areas. The bottom panel shows results for river segments located within IUCN-categorized protected areas. The figure displays the top 15 primary mining commodities with the highest estimated contribution in each panel. Box sizes represent each commodity's share of the total potentially contaminated river length in the respective spatial context. Because multiple mining commodities may influence a single river segment, values are not mutually exclusive. To improve clarity, the figure excludes the Unknown category (representing 81,137 km in the top panel and 38,732 km in the bottom panel), where data on the primary commodity of the mine were unavailable.*

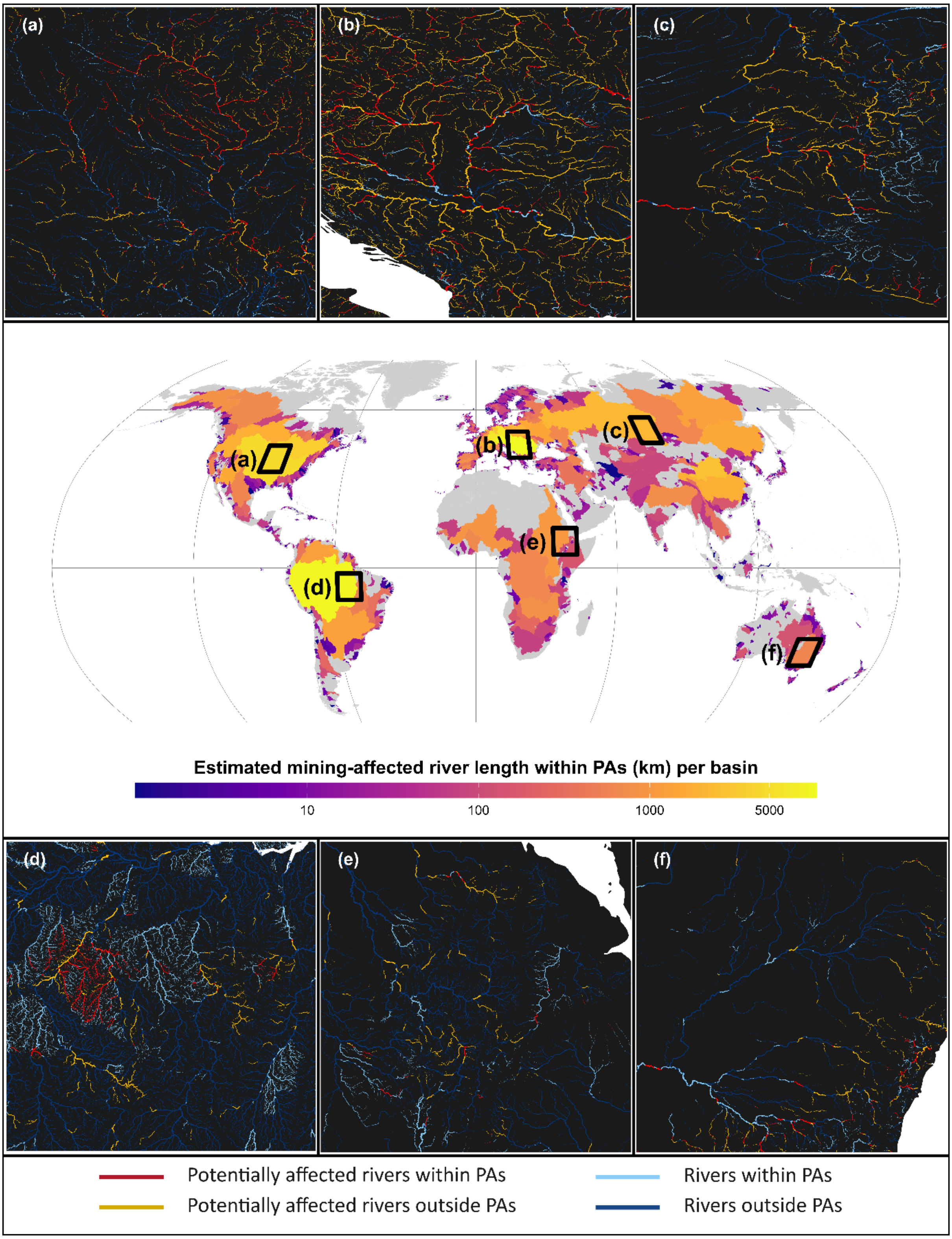

Figure S10. *Distribution of rivers potentially exposed to mining-related contamination within IUCN-categorized protected areas (PAs), based on the severe modeling assumption. The middle panel presents a world map showing the total river length potentially exposed to mining impacts within PAs, aggregated by hydrological basin. To enhance visualization, the scale is* log_{10}*-transformed, with labels expressed in kilometers for easier interpretation. The bounding boxes in the main map indicate the locations of the insets shown in the top (a–c) and bottom (d–f) panels. These insets provide detailed views of hydrological basins in each continent with a high estimated extent of overlap between potentially exposed river reaches and PAs. In these zoomed-in views, river line width is proportional to the annual average natural river discharge. For visualization purposes, only river segments with an annual average discharge greater than 0.5* m^3/s *are displayed.*

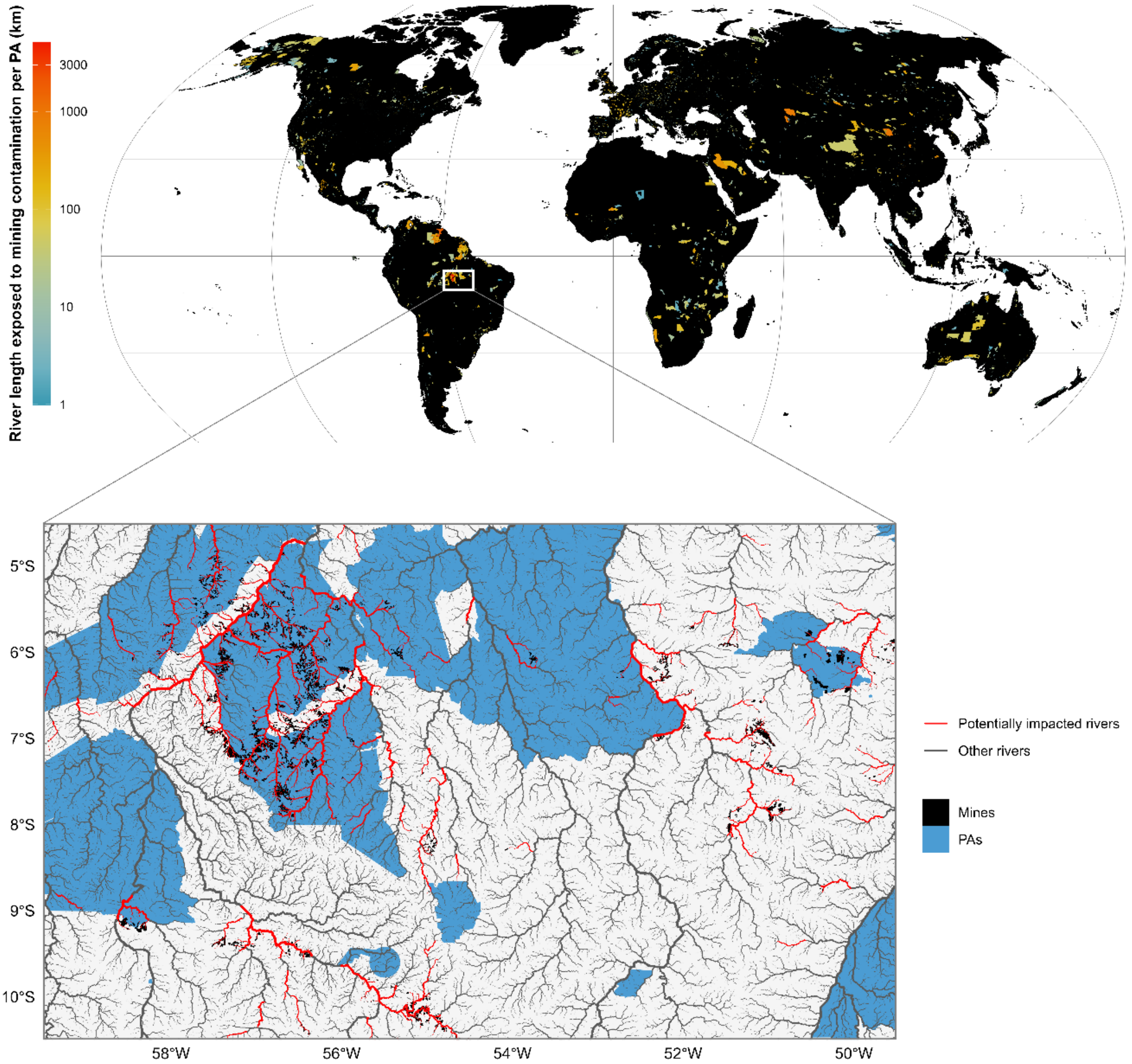

***Figure S11**. Global patterns of estimated river exposure to mining-related contamination across IUCN-categorized protected areas (PAs). The main map shows PAs colored by the total length of rivers potentially exposed to mining contamination within each area. The color gradient represents $\log_{10}$-transformed river length within each polygon under the severe modeling assumption, with values expressed in kilometers for interpretability. The inset highlights a region in Brazil PAs with high projected exposure. Black polygons indicate mine footprints, the majority of which are gold mines (primary commodity). For visualization purposes, only rivers with an annual average discharge greater than 0.5 m^3/s are shown. Potentially exposed rivers are highlighted in red, while remaining rivers are shown in grey.*

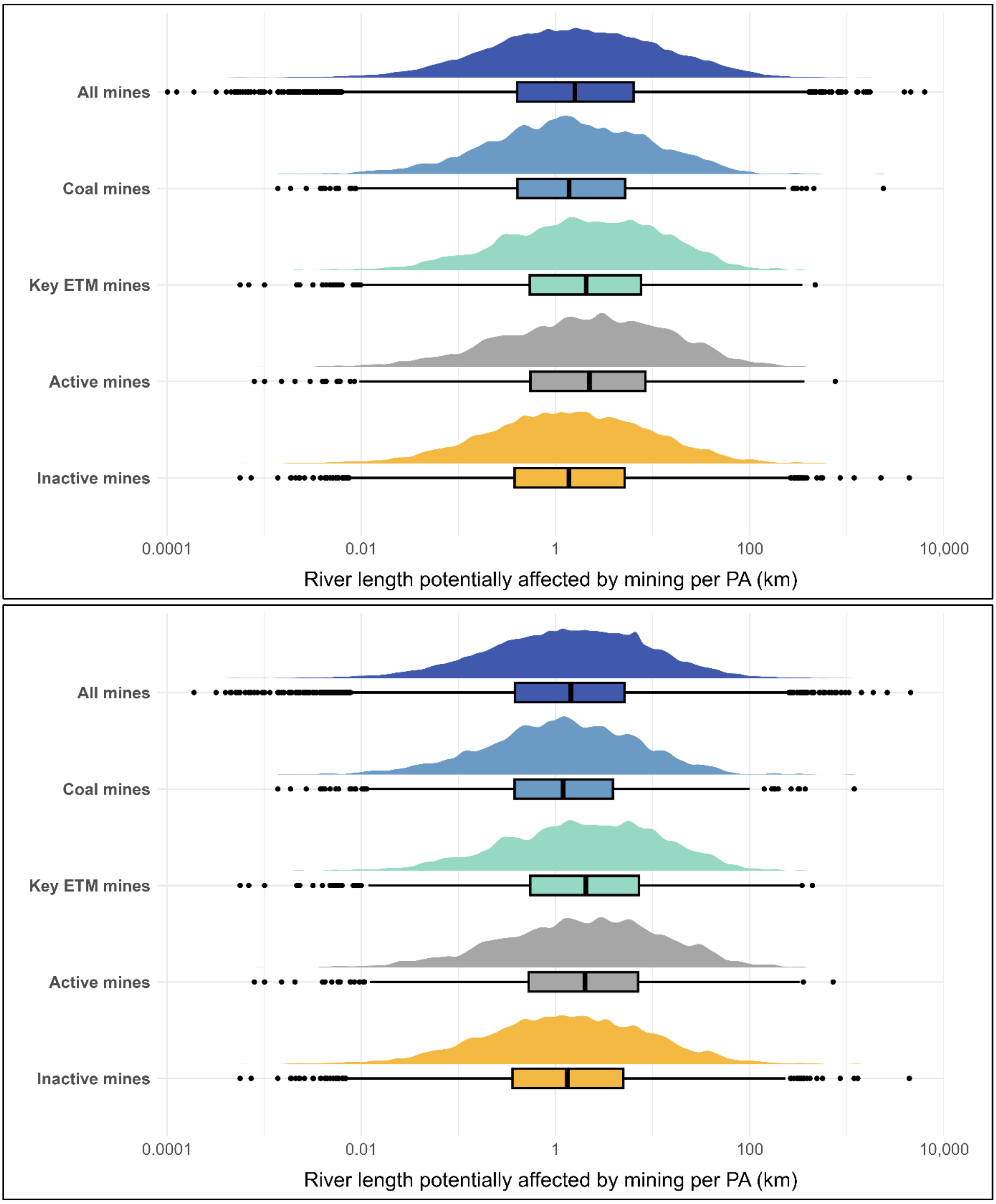

Figure S12. *Raincloud plots showing the total length (in km) of river segments potentially exposed to mining-related contamination within individual protected areas (PAs), under the severe (upper panel) and moderate (lower panel) modeling assumptions. Values are log*$_{10}$*-transformed to account for the wide range of affected river lengths but are presented in kilometers to aid interpretation.*

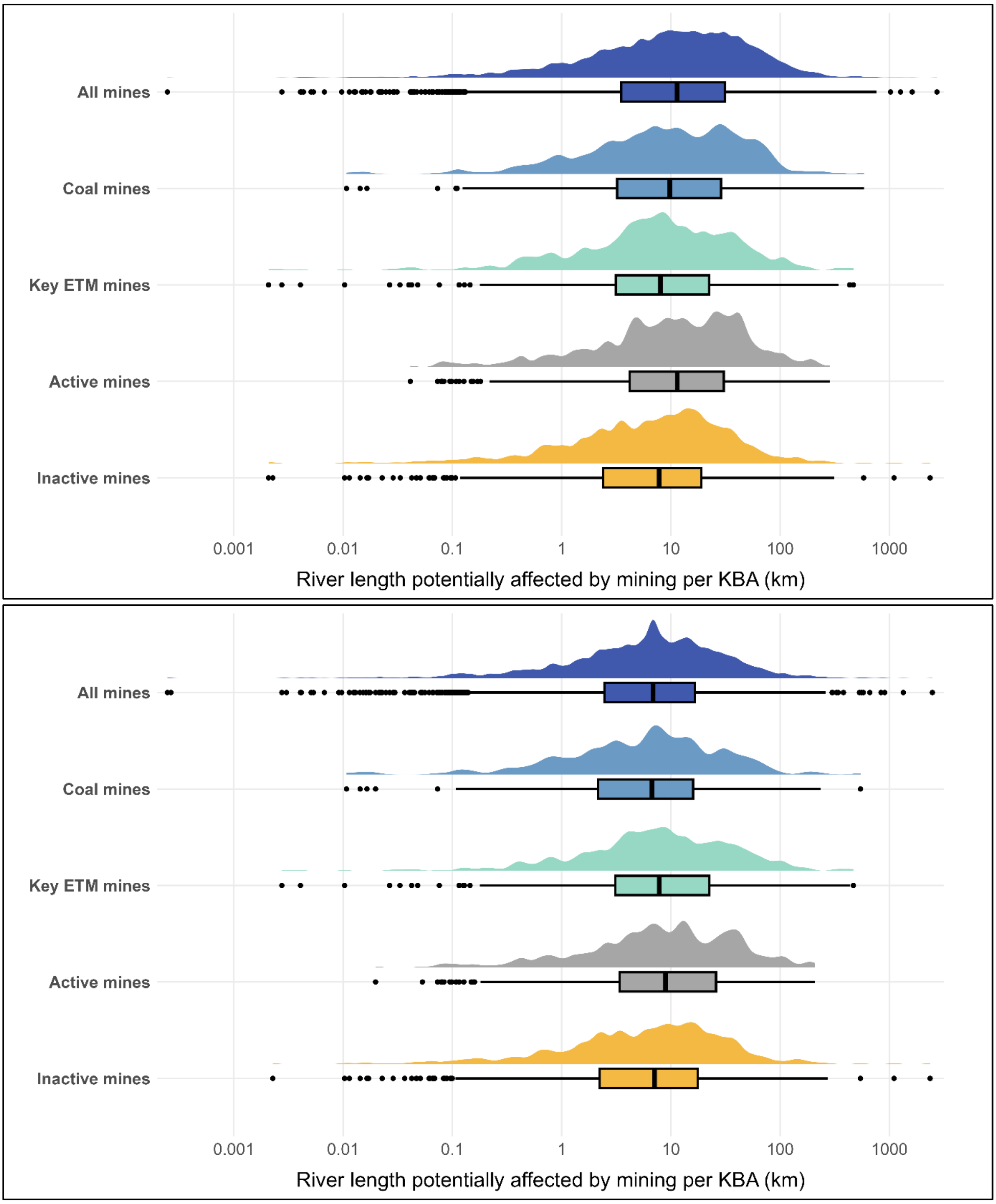

***Figure S13**. Raincloud plots showing the total length (in km) of river segments potentially exposed to mining-related contamination within individual Key Biodiversity Areas (KBAs), under the severe (upper panel) and moderate (lower panel) modeling assumptions. Values are $\log_{10}$-transformed to account for the wide range of affected river lengths but are presented in kilometers to aid interpretation.*

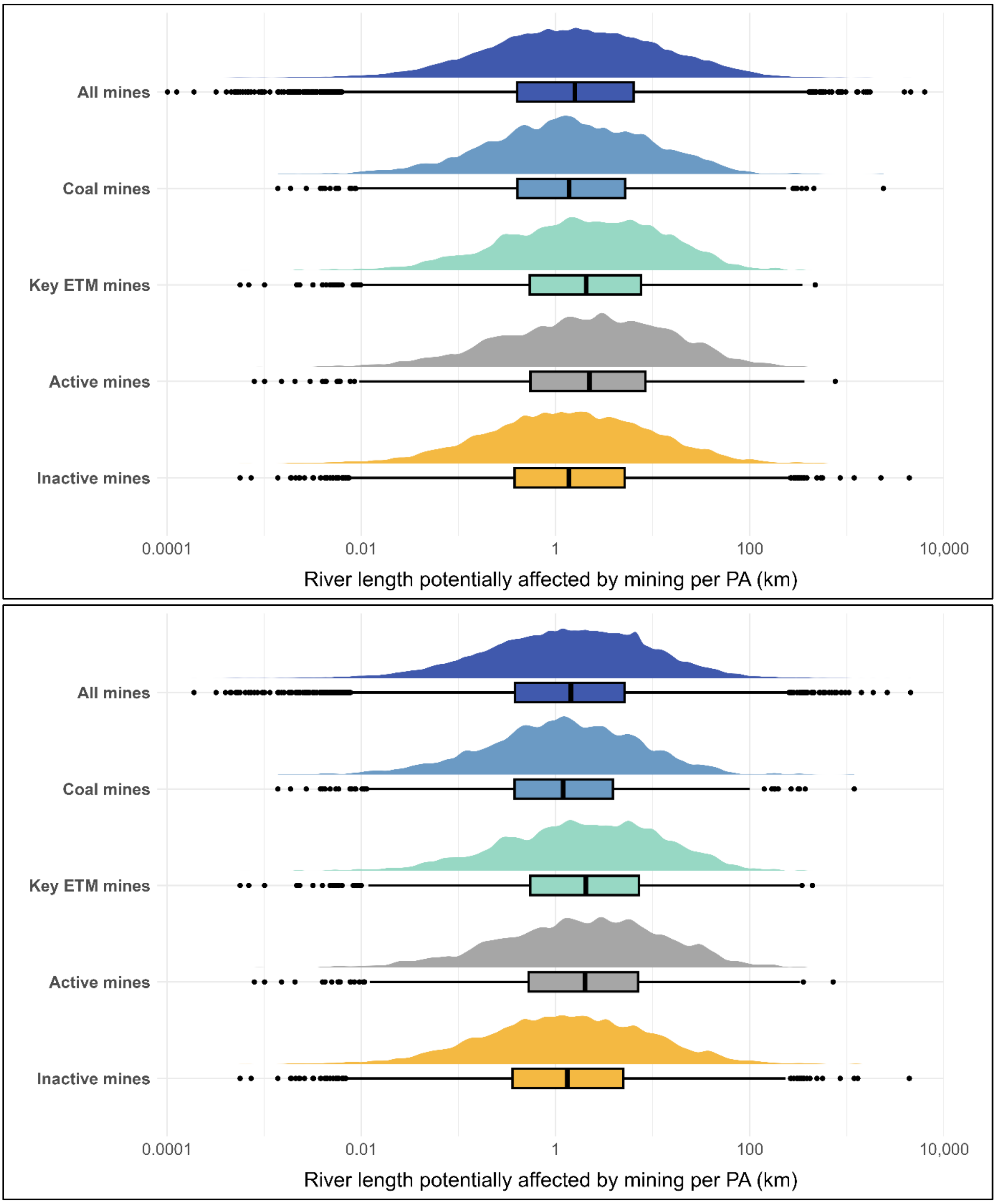

***Figure S14**. Raincloud plots showing the total length (in km) of river segments potentially exposed to mining-related contamination within individual protected areas (PAs) with assigned IUCN management categories, under the severe (upper panel) and moderate (lower panel) modeling assumptions. Values are log_{10}-transformed to account for the wide range of affected river lengths but are presented in kilometers to aid interpretation.*

SI Tables

Table S1. *List of unique known primary commodities in the global mining dataset used in this study.*

**Primary mining commodities**		
Aluminium	Heavy Mineral Sands	Platinum Group Element
Antimony	Iron	Potash
Chromite	Kaolin	Rare Earth Element
Chromium	Lead	Silver
Coal	Limestone	Tantalum
Cobalt	Lithium	Tin
Copper	Manganese	Titanium
Diamonds	Molybdenum	Tungsten
Dolomite	Nepheline	Uranium
Ferrochrome	Nickel	Vanadium
Fluorspar	Niobium	Zinc
Gold	Phosphate	Zircon
Graphite		

Table S2. *Mapping of original mine-property statuses to standardized activity categories. The original reported statuses were retrieved from (2, 17, 18) and harmonized into four categories: Active, Inactive, Planned, and Unknown.*

**Original activity status**	**Simplified activity status**
Active	Active
Advanced exploration	Planned
Cancelled	Inactive
Care and maintenance	Inactive
Closed	Inactive
Construction	Planned
Exploration	Planned
In development	Planned
Inactive	Inactive
Mothballed	Inactive
On hold awaiting financing	Inactive
On hold awaiting higher prices	Inactive
Operating	Active
Project	Planned
Proposed	Planned
Rehabilitation	Inactive
Shelved	Inactive
Temporarily on hold	Inactive
Under construction	Planned
Under litigation	Inactive
NA	Unknown

Table S3. *Summary statistics for mapped mining areas with known and unknown primary commodities. The table reports summary statistics on the area (in hectares) of individual mining polygons with and without known primary commodity information in the integrated global mining dataset.*

	Area of mines with known commodities (hectares)	Area of mines with unknown commodities (hectares)
Minimum	2.2×10^{-3}	2.4×10^{-3}
Maximum	491,000	255,000
Mean	112	30
Median	6.1	3.6
SD	1,880	834
IQR	25.9	11.9

Table S4. *Mapped mining area by commodity information availability and country. The table summarizes the total mapped mining area by country in hectares, disaggregated by whether the primary commodity is known or unknown. The 'share of unknown area' indicates the percentage of each country's total mapped mining area for which commodity information is missing, while the 'contribution to total unknown area' reflects the country's share of the global total mapped mining area lacking commodity data. Country definitions follow the administrative units in the Natural Earth "Admin 0 – Countries" dataset* (*19*)*, which includes 258 entities. Countries are listed in descending order by the area with unknown commodity information.*

Country	Mapped area with known commodities (ha)	Mapped area with unknown commodities (ha)	Total mapped mining area (ha)	Share of unknown area (%)	Contribution to total unknown area (%)
Russia	1179388	590082	1769470	33.30	17.42
United States of America	1181930	357193	1539123	23.20	10.54
Peru	112257	266501	378758	70.40	7.87
China	1287802	264909	1552711	17.10	7.82
Myanmar	66907	154633	221540	69.80	4.56
Brazil	562933	116562	679495	17.20	3.44
India	277749	109951	387700	28.40	3.25
Australia	1094511	98588	1193099	8.30	2.91
Indonesia	771721	94891	866612	10.90	2.80
Saudi Arabia	13113	94162	107276	87.80	2.78
Argentina	166681	94100	260780	36.10	2.78
Canada	1056246	92058	1148304	8.00	2.72
France	32175	70244	102419	68.60	2.07
Germany	100223	49979	150202	33.30	1.48
Spain	32512	43429	75941	57.20	1.28
Kazakhstan	230571	43416	273987	15.80	1.28
Guyana	202376	40139	242515	16.60	1.18
Estonia	0	36736	36736	100.00	1.08
Iran	39765	35840	75604	47.40	1.06
Turkey	79269	34986	114255	30.60	1.03
Italy	11806	34751	46557	74.60	1.03
Finland	39708	29161	68869	42.30	0.86
Ukraine	113732	29021	142753	20.30	0.86
Iraq	887	27610	28497	96.90	0.82
Venezuela	124382	26988	151370	17.80	0.80

Poland	53479	26109	79589	32.80	0.77
United Kingdom	36534	21868	58403	37.40	0.65
Chile	518790	21783	540573	4.00	0.64
Egypt	7832	21672	29504	73.50	0.64
South Africa	411869	17013	428881	4.00	0.50
Lithuania	0	16899	16899	100.00	0.50
Libya	0	16308	16308	100.00	0.48
Sweden	20588	16087	36675	43.90	0.47
Mexico	102610	15745	118355	13.30	0.46
Algeria	4588	14525	19113	76.00	0.43
Colombia	94840	12830	107670	11.90	0.38
Japan	1042	12430	13472	92.30	0.37
Latvia	0	11887	11887	100.00	0.35
Bulgaria	32405	11620	44025	26.40	0.34
Romania	22181	11559	33740	34.30	0.34
Mongolia	94657	9873	104530	9.40	0.29
Azerbaijan	2397	9752	12149	80.30	0.29
Sudan	37870	9310	47180	19.70	0.27
Namibia	43685	9077	52762	17.20	0.27
United Arab Emirates	427	9058	9485	95.50	0.27
Syria	0	9033	9033	100.00	0.27
Greece	27873	8955	36828	24.30	0.26
Israel	39515	8726	48241	18.10	0.26
Portugal	5057	8392	13450	62.40	0.25
Norway	5151	8307	13458	61.70	0.25
Thailand	15801	7598	23399	32.50	0.22
Morocco	45967	7183	53150	13.50	0.21
Côte d'Ivoire	4705	6959	11663	59.70	0.21
Malaysia	14834	6865	21699	31.60	0.20
Belarus	2832	6615	9447	70.00	0.20
Qatar	0	6599	6599	100.00	0.19
Hungary	9644	6047	15691	38.50	0.18
Bolivia	32859	5617	38476	14.60	0.17
Belgium	1373	5596	6969	80.30	0.17
Czechia	26343	5290	31634	16.70	0.16
Vietnam	30274	5001	35276	14.20	0.15
Austria	7029	4879	11908	41.00	0.14
Denmark	0	4822	4822	100.00	0.14
New Zealand	13888	4628	18516	25.00	0.14
Philippines	32785	4367	37152	11.80	0.13
Switzerland	367	4291	4658	92.10	0.13
Pakistan	4591	3763	8355	45.00	0.11
Ireland	10053	3619	13671	26.50	0.11
Zimbabwe	31069	3614	34683	10.40	0.11
Suriname	215033	3574	218607	1.60	0.11
Slovakia	2458	3425	5883	58.20	0.10

Dem. Rep. Congo	47266	3213	50479	6.40	0.09
Lebanon	0	3128	3128	100.00	0.09
Croatia	11	3056	3067	99.60	0.09
Guinea	29456	2955	32411	9.10	0.09
Jordan	32735	2914	35649	8.20	0.09
Burkina Faso	19296	2914	22210	13.10	0.09
Tunisia	9169	2849	12018	23.70	0.08
Uzbekistan	48010	2818	50828	5.50	0.08
Cuba	14065	2635	16700	15.80	0.08
Nepal	0	2490	2490	100.00	0.07
Ghana	199671	2456	202127	1.20	0.07
New Caledonia	27472	2436	29908	8.10	0.07
Serbia	15015	2356	17370	13.60	0.07
Niger	13885	2350	16235	14.50	0.07
Armenia	6912	2324	9236	25.20	0.07
Angola	34815	2118	36933	5.70	0.06
Bosnia and Herz.	9031	1996	11027	18.10	0.06
Taiwan	7	1948	1955	99.60	0.06
Senegal	7682	1939	9621	20.20	0.06
Moldova	0	1889	1889	100.00	0.06
Ecuador	6759	1858	8616	21.60	0.05
Central African Rep.	11	1856	1867	99.40	0.05
Nicaragua	1975	1707	3683	46.40	0.05
Tanzania	14473	1545	16019	9.60	0.05
Ethiopia	1696	1477	3174	46.60	0.04
Cameroon	959	1446	2405	60.10	0.04
North Macedonia	6175	1436	7611	18.90	0.04
Palestine	0	1316	1316	100.00	0.04
Mali	22171	1287	23458	5.50	0.04
Iceland	295	1215	1510	80.40	0.04
Nigeria	2475	1182	3657	32.30	0.03
Bangladesh	130	1133	1263	89.70	0.03
Turkmenistan	1307	1122	2429	46.20	0.03
Dominican Rep.	3316	1089	4406	24.70	0.03
Madagascar	4449	1060	5509	19.20	0.03
Oman	22008	1050	23057	4.60	0.03
Mozambique	16675	1045	17720	5.90	0.03
Laos	6790	1044	7834	13.30	0.03
North Korea	6501	1039	7540	13.80	0.03
Botswana	33953	1024	34977	2.90	0.03
Liberia	1752	950	2702	35.10	0.03
Paraguay	384	920	1304	70.50	0.03
Mauritania	13592	857	14449	5.90	0.03
Kenya	2640	854	3494	24.40	0.03
South Korea	4641	843	5484	15.40	0.02
Slovenia	578	836	1414	59.10	0.02

Cambodia	149	795	944	84.20	0.02
Sierra Leone	12121	730	12851	5.70	0.02
Netherlands	206	724	929	77.90	0.02
Puerto Rico	0	718	718	100.00	0.02
Kosovo	3172	675	3846	17.50	0.02
Papua New Guinea	12766	648	13415	4.80	0.02
Zambia	55889	617	56506	1.10	0.02
Bir Tawil	0	555	555	100.00	0.02
Cyprus	1635	548	2183	25.10	0.02
Congo	1449	537	1987	27.00	0.02
Costa Rica	505	509	1014	50.20	0.02
Uruguay	1416	498	1914	26.00	0.01
Gabon	5964	490	6455	7.60	0.01
N. Cyprus	182	488	670	72.90	0.01
Kuwait	0	467	467	100.00	0.01
Somaliland	61	464	525	88.40	0.01
Indian Ocean Ter.	0	434	434	100.00	0.01
Tajikistan	6311	432	6743	6.40	0.01
Uganda	430	431	861	50.10	0.01
Afghanistan	1388	399	1787	22.30	0.01
Georgia	2998	391	3388	11.50	0.01
Albania	2539	390	2930	13.30	0.01
Kyrgyzstan	13297	378	13676	2.80	0.01
Bhutan	0	365	365	100.00	0.01
Chad	20	364	384	94.80	0.01
Malta	0	341	341	100.00	0.01
Yemen	0	316	316	100.00	0.01
Somalia	10	315	324	97.00	0.01
Sri Lanka	1425	301	1727	17.40	0.01
Benin	24	282	306	92.20	0.01
Panama	3008	265	3274	8.10	0.01
Togo	1455	260	1714	15.10	0.01
S. Sudan	0	249	249	100.00	0.01
Trinidad and Tobago	0	234	234	100.00	0.01
Haiti	337	230	567	40.60	0.01
Belize	0	230	230	100.00	0.01
Hong Kong	0	210	210	100.00	0.01
Eritrea	1226	204	1430	14.30	0.01
Burundi	0	185	185	100.00	0.01
Djibouti	0	178	178	100.00	0.01
Cabo Verde	0	173	173	100.00	0.01
Montenegro	1268	151	1419	10.60	0.00
Lesotho	1394	145	1538	9.40	0.00
Kiribati	0	142	142	100.00	0.00
Guatemala	1383	129	1512	8.50	0.00
Barbados	0	125	125	100.00	0.00

Brunei	0	122	122	100.00	0.00
Jamaica	4344	122	4466	2.70	0.00
Falkland Is.	0	113	113	100.00	0.00
Mauritius	0	113	113	100.00	0.00
Bahamas	0	112	112	100.00	0.00
Åland	0	111	111	100.00	0.00
Eq. Guinea	0	110	110	100.00	0.00
Isle of Man	0	109	109	100.00	0.00
Gambia	0	107	107	100.00	0.00
Guinea-Bissau	11	103	115	90.10	0.00
Comoros	0	97	97	100.00	0.00
Fiji	440	93	534	17.50	0.00
Guam	0	89	89	100.00	0.00
Malawi	542	88	630	14.00	0.00
Luxembourg	492	81	573	14.10	0.00
Dhekelia	0	71	71	100.00	0.00
St. Kitts and Nevis	0	70	70	100.00	0.00
El Salvador	59	69	127	53.90	0.00
Rwanda	169	68	237	28.80	0.00
Timor-Leste	0	66	66	100.00	0.00
Faeroe Is.	0	63	63	100.00	0.00
Singapore	0	60	60	100.00	0.00
Tonga	0	59	59	100.00	0.00
Saint Lucia	0	59	59	100.00	0.00
Curaçao	0	47	47	100.00	0.00
Nauru	0	46	46	100.00	0.00
Grenada	0	43	43	100.00	0.00
Antigua and Barb.	0	37	37	100.00	0.00
Cayman Is.	0	35	35	100.00	0.00
N. Mariana Is.	0	31	31	100.00	0.00
U.S. Virgin Is.	0	30	30	100.00	0.00
Anguilla	0	30	30	100.00	0.00
Dominica	0	29	29	100.00	0.00
Jersey	0	29	29	100.00	0.00
Greenland	36	29	65	44.30	0.00
Monaco	0	28	28	100.00	0.00
Honduras	1098	28	1126	2.50	0.00
Bermuda	0	24	24	100.00	0.00
São Tomé and Principe	0	22	22	100.00	0.00
St-Martin	0	22	22	100.00	0.00
Baikonur	0	22	22	100.00	0.00
Samoa	0	20	20	100.00	0.00
St. Pierre and Miquelon	0	18	18	100.00	0.00
Guernsey	0	17	17	100.00	0.00
Vanuatu	0	16	16	100.00	0.00
Liechtenstein	0	11	11	100.00	0.00

Turks and Caicos Is.	0	9	9	100.00	0.00
Palau	0	7	7	100.00	0.00
Cook Is.	0	5	5	100.00	0.00
British Virgin Is.	0	4	4	100.00	0.00
Montserrat	0	3	3	100.00	0.00
Cyprus U.N. Buffer Zone	487	3	490	0.60	0.00
eSwatini	119	3	122	2.40	0.00
Fr. Polynesia	0	3	3	100.00	0.00
San Marino	0	2	2	100.00	0.00
Fr. S. Antarctic Lands	0	2	2	100.00	0.00
Norfolk Island	0	2	2	100.00	0.00
American Samoa	0	2	2	100.00	0.00
Andorra	0	1	1	100.00	0.00
Macao	0	1	1	100.00	0.00
Antarctica	0	0	0	100.00	0.00
Aruba	141	0	141	0.00	0.00
Niue	2	0	2	0.00	0.00
Solomon Is.	594	0	594	0.00	0.00

Table S5. *Count of mapped mining polygons by commodity information availability and country. The table summarizes the number of mapped mining polygons by country, disaggregated by whether the primary commodity is known or unknown. The 'share of unknown polygons' indicates the percentage of each country's total mapped polygons for which commodity information is missing. Country definitions follow the administrative units in the Natural Earth "Admin 0 – Countries" dataset (19), which includes 258 entities. Countries are listed in descending order by the number of polygons with unknown commodity information.*

Country	Mapped polygons (count)	Polygons with unknown commodities (count)	Share of unknown polygons (%)
China	37198	10362	27.9
United States of America	16451	10073	61.2
Russia	13607	9379	68.9
Canada	16899	8034	47.5
Italy	5629	4473	79.5
Germany	8537	4377	51.3
Brazil	6382	3873	60.7
France	4719	3845	81.5
Algeria	3525	3383	96
Finland	4675	2752	58.9
Spain	3714	2687	72.3
Turkey	4266	2605	61.1
Norway	3388	2467	72.8
United Kingdom	5734	2444	42.6
India	5323	2297	43.2
Poland	2637	1964	74.5
Ukraine	3647	1609	44.1
Sweden	2070	1549	74.8
Argentina	2455	1429	58.2

Australia	4954	1388	28
Central African Rep.	1303	1288	98.8
Iran	1490	1274	85.5
Burkina Faso	2097	1081	51.5
Switzerland	1028	933	90.8
Nepal	909	908	99.9
Austria	2720	825	30.3
Belarus	843	799	94.8
Mexico	2303	749	32.5
Iraq	705	701	99.4
Portugal	898	657	73.2
Greece	1252	656	52.4
Saudi Arabia	715	632	88.4
Japan	659	597	90.6
Colombia	1403	594	42.3
Dem. Rep. Congo	1097	582	53.1
Czechia	1018	559	54.9
South Africa	2345	527	22.5
Niger	591	508	86
Hungary	759	502	66.1
Morocco	655	484	73.9
Indonesia	2526	460	18.2
New Zealand	818	438	53.5
Lithuania	436	436	100
Croatia	425	424	99.8
Romania	565	423	74.9
Denmark	417	417	100
Chile	1389	366	26.3
Kazakhstan	1160	366	31.6
Bulgaria	586	356	60.8
Ireland	950	337	35.5
Belgium	373	335	89.8
Cameroon	360	330	91.7
Latvia	329	329	100
Syria	319	319	100
Slovakia	906	317	35
Philippines	666	282	42.3
Ethiopia	320	268	83.8
Lebanon	262	262	100
Egypt	304	257	84.5
Thailand	323	257	79.6
Myanmar	362	247	68.2
Iceland	269	239	88.8
Bolivia	494	233	47.2
Vietnam	510	225	44.1
Tunisia	257	213	82.9

Estonia	211	211	100
Peru	1297	204	15.7
Liberia	306	203	66.3
Bosnia and Herz.	373	200	53.6
Cuba	308	194	63
Sierra Leone	766	192	25.1
Armenia	369	178	48.2
Senegal	222	178	80.2
Uganda	208	175	84.1
Paraguay	215	173	80.5
Malaysia	349	165	47.3
Mauritania	205	158	77.1
Mongolia	711	153	21.5
North Korea	600	150	25
Mozambique	228	149	65.4
Serbia	320	148	46.2
United Arab Emirates	154	148	96.1
Lesotho	161	147	91.3
Zimbabwe	682	144	21.1
Belize	142	142	100
Pakistan	294	142	48.3
Moldova	139	139	100
Libya	137	137	100
Venezuela	273	134	49.1
Guyana	463	132	28.5
Angola	678	129	19
Ecuador	261	121	46.4
Jordan	213	121	56.8
Tanzania	376	119	31.6
Slovenia	251	118	47
Uzbekistan	229	116	50.7
Guinea	502	112	22.3
Kosovo	300	108	36
Taiwan	103	102	99
Ghana	1509	99	6.6
Azerbaijan	134	98	73.1
Kenya	125	95	76
Sri Lanka	228	95	41.7
Nigeria	159	90	56.6
Israel	113	89	78.8
Nicaragua	124	87	70.2
North Macedonia	137	87	63.5
Haiti	92	81	88
Somalia	80	79	98.8
Albania	245	78	31.8
Namibia	377	78	20.7

Somaliland	79	77	97.5
Mali	185	76	41.1
Sudan	237	76	32.1
Chad	74	72	97.3
Afghanistan	122	69	56.6
Malta	69	69	100
Suriname	970	68	7
Madagascar	128	64	50
South Korea	195	61	31.3
Georgia	137	60	43.8
Netherlands	67	59	88.1
Zambia	295	58	19.7
Benin	59	56	94.9
Panama	85	56	65.9
Palestine	54	54	100
Papua New Guinea	99	52	52.5
Côte d'Ivoire	79	51	64.6
Cabo Verde	50	50	100
Uruguay	83	50	60.2
Laos	108	49	45.4
Dominican Rep.	69	48	69.6
Puerto Rico	47	47	100
Botswana	209	44	21.1
S. Sudan	42	42	100
Costa Rica	65	39	60
Cambodia	51	38	74.5
New Caledonia	392	37	9.4
Montenegro	58	35	60.3
Isle of Man	34	34	100
Congo	79	33	41.8
Kyrgyzstan	151	33	21.9
Oman	240	33	13.8
Tajikistan	98	32	32.7
Burundi	31	31	100
Bir Tawil	29	29	100
Togo	34	29	85.3
Yemen	29	29	100
N. Cyprus	30	28	93.3
Bhutan	27	27	100
Gabon	53	25	47.2
Rwanda	42	24	57.1
Cyprus	76	23	30.3
Bangladesh	25	22	88
Guinea-Bissau	25	22	88
Tonga	22	22	100
Indian Ocean Ter.	21	21	100

Jamaica	185	21	11.4
Faeroe Is.	18	18	100
Qatar	18	18	100
Guernsey	17	17	100
Bahamas	13	13	100
Falkland Is.	13	13	100
Turkmenistan	16	13	81.2
Vanuatu	13	13	100
Eq. Guinea	12	12	100
Gambia	12	12	100
Malawi	31	12	38.7
Bermuda	11	11	100
Djibouti	11	11	100
Mauritius	11	11	100
Dominica	10	10	100
Guatemala	38	10	26.3
Luxembourg	40	10	25
St. Kitts and Nevis	10	10	100
Hong Kong	9	9	100
Saint Lucia	9	9	100
Timor-Leste	9	9	100
Samoa	8	8	100
Barbados	7	7	100
Greenland	9	7	77.8
Guam	7	7	100
Brunei	6	6	100
Honduras	24	6	25
Liechtenstein	6	6	100
Nauru	6	6	100
São Tomé and Principe	6	6	100
Trinidad and Tobago	5	5	100
Åland	5	5	100
Antigua and Barb.	4	4	100
El Salvador	9	4	44.4
Eritrea	13	4	30.8
Fr. Polynesia	4	4	100
Grenada	4	4	100
Jersey	4	4	100
N. Mariana Is.	4	4	100
eSwatini	8	4	50
Antarctica	3	3	100
Cayman Is.	3	3	100
Comoros	3	3	100
Cook Is.	3	3	100
Dhekelia	3	3	100
Fiji	23	3	13

Kuwait	3	3	100
Montserrat	3	3	100
Singapore	3	3	100
U.S. Virgin Is.	3	3	100
British Virgin Is.	2	2	100
Curaçao	2	2	100
Fr. S. Antarctic Lands	2	2	100
Palau	2	2	100
St. Pierre and Miquelon	2	2	100
American Samoa	1	1	100
Andorra	1	1	100
Anguilla	1	1	100
Baikonur	1	1	100
Cyprus U.N. Buffer Zone	3	1	33.3
Kiribati	1	1	100
Macao	1	1	100
Monaco	1	1	100
Norfolk Island	1	1	100
San Marino	1	1	100
St-Martin	1	1	100
Turks and Caicos Is.	1	1	100
Aruba	26	0	0
Niue	4	0	0
Solomon Is.	3	0	0

Descriptions of Datasets S1 to S5

Dataset S1. Summary of river length potentially affected by mining across all mine subsets and modeling assumptions. The Excel file contains four main sheets, each corresponding to a different assumption used to model downstream contaminant dispersion from mines: moderate, severe, lower-bound (uniform 6.5 km), and upper-bound (uniform 45.6 km). For each mine subset (defined by commodity type, operational status, or data completeness), the dataset reports the total length of potentially affected rivers (in km), as well as the portions overlapping with Protected Areas (PAs), Key Biodiversity Areas (KBAs), their overlap, and Conservation Priority Areas (CPAs). PA-related metrics are reported for the full set of PAs and for a subset containing only sites with assigned IUCN management categories. The table provides both absolute and proportional exposure values, including: (i) the percentage of river length within each conservation designation that is potentially affected, and (ii) the share of total affected rivers that fall within each designation. Complete definitions of all abbreviations, mine subsets, and column variables are provided within the file.

Dataset S2. Estimated proportion of potentially affected river length attributable to the current clean energy demand for each key energy transition mineral (ETM) mining subset, under four downstream contamination modeling assumptions (moderate, severe, lower-bound, upper-bound). For each mine subset, the dataset reports the percentage of potentially affected river length linked to global clean energy demand in 2023, based on mineral-specific demand shares reported by the International Energy Agency (*16*). These percentages are shown at the global level, and separately for river segments located within Protected Areas (PAs), Key Biodiversity Areas (KBAs), their overlap, and Conservation Priority Areas (CPAs). PA-related values are provided for the full set of PAs and for a subset containing only sites with assigned IUCN management categories. For mines extracting multiple key ETMs, demand percentages were averaged across minerals prior to scaling. All definitions and assumptions are documented within the file.

Dataset S3. Summary of river length potentially affected by mining, disaggregated by hydrological basin. The Excel file contains four main sheets, each corresponding to a different assumption used to model downstream contaminant dispersion from mines: moderate, severe, lower-bound (uniform 6.5 km), and upper-bound (uniform 45.6 km). Within each sheet, results are summarized at the hydrological basin level for the full dataset of mines included in the study. For each basin, the dataset reports the total length of potentially affected rivers (in km), as well as the portion of that length overlapping with Protected Areas (PAs), Key Biodiversity Areas (KBAs), their overlap, and Conservation Priority Areas (CPAs). PA-related metrics are reported for the full set of PAs and for a subset containing only sites with assigned IUCN management categories. The table provides both absolute and proportional exposure values, including: (i) the percentage of river length within each conservation designation that is potentially affected within a basin, and (ii) the share of total affected river length in each basin that falls within each designation. Complete definitions of all abbreviations and column variables are provided within the file.

Dataset S4. Summary of river lengths potentially affected by mining, reported at the Key Biodiversity Area (KBA) level. The Excel file includes four sheets corresponding to different assumptions for

modeling downstream contaminant dispersion: moderate, severe, lower-bound (uniform 6.5 km), and upper-bound (uniform 45.6 km). Each sheet summarizes, for every KBA, the total length of potentially affected rivers (in km) and the percentage of the KBA's river network that is potentially affected.

Dataset S5. Summary of river lengths potentially affected by mining, reported at the Protected Area (PA) level. The Excel file includes four sheets corresponding to different assumptions for modeling downstream contaminant dispersion: moderate, severe, lower-bound (uniform 6.5 km), and upper-bound (uniform 45.6 km). Each sheet summarizes, for every PA, the total length of potentially affected rivers (in km) and the percentage of the PA's river network that is potentially affected.

SI references

1. V. Maus, A data-driven approach to mapping global commodity-specific mining land-use. *J Clean Prod* 540, 147437 (2026).

2. S&P Global Market Intelligence, S&P Capital IQ Pro database. [Preprint] (2024). https://www.spglobal.com/market-intelligence/en/solutions/products/sp-capital-iq-pro.

3. V. Maus, T. T. Werner, Impacts for half of the world's mining areas are undocumented. *Nature* 625, 26–29 (2024).

4. G. P. Asner, R. Tupayachi, Accelerated losses of protected forests from gold mining in the Peruvian Amazon. *Environmental Research Letters* 12 (2017).

5. G. P. Asner, R. Llactayo, R. Tupayachi, E. R. Luna, Elevated rates of gold mining in the Amazon revealed through high-resolution monitoring. *Proc Natl Acad Sci U S A* 110, 18454–18459 (2013).

6. J. Siqueira-Gay, L. E. Sánchez, The outbreak of illegal gold mining in the Brazilian Amazon boosts deforestation. *Reg Environ Change* 21 (2021).

7. L. Cortinhas Ferreira Neto, C. G. Diniz, R. V. Maretto, C. Persello, M. L. Silva Pinheiro, M. C. Castro, L. W. Rodrigues Sadeck, A. Fernandes Filho, J. Cansado, A. A. de Almeida Souza, J. Pinto Feitosa, D. C. Santos, M. Adami, P. W. M. Souza-Filho, A. Stein, A. Biehl, A. Klautau, Uncontrolled Illegal Mining and Garimpo in the Brazilian Amazon. *Nat Commun* 15 (2024).

8. K. J. LaJeunesse Connette, G. Connette, A. Bernd, P. Phyo, K. H. Aung, Y. L. Tun, Z. M. Thein, N. Horning, P. Leimgruber, M. Songer, Assessment of Mining Extent and Expansion in Myanmar Based on Freely-Available Satellite Imagery. *Remote Sens (Basel)* 8, 912 (2016).

9. T. Osawa, Y. Hatsukawa, Artisanal and small-scale gold mining in Myanmar: Preliminary research for environmental mercury contamination. *International Journal of Human Culture Studies*, 221–230 (2015).

10. A. Knizhnikov, E. Shvarts, L. Ametistova, A. Pakhalov, N. Rozhkova, D. Yudaeva, Environmental transparency of Russian mining and metal companies: Evidence from independent ranking system. *Extr Ind Soc* 8, 100937 (2021).

11. A. Grajal-Puche, E. M. Driver, C. R. Propper, Review: Abandoned mines as a resource or liability for wildlife. *Science of The Total Environment* 921, 171017 (2024).

12. K. P. Young, B. R. Murray, L. J. Martin, M. L. Murray, Lost but Not Forgotten: Identifying Unmapped and Unlisted Environmental Hazards including Abandoned Mines. *Sustainability* 13, 11011 (2021).

13. L. Shen, T. Dai, A. J. Gunson, Small-scale mining in China: Assessing recent advances in the policy and regulatory framework. *Resources Policy* 34, 150–157 (2009).

14. S. Linke, B. Lehner, C. O. Dallaire, J. Ariwi, G. Grill, M. Anand, P. Beames, V. Burchard-Levine, S. Maxwell, H. Moidu, F. Tan, M. Thieme, Global hydro-environmental sub-basin and river reach characteristics at high spatial resolution. *Sci Data* 6, 283 (2019).

15. M. G. Macklin, C. J. Thomas, A. Mudbhatkal, P. A. Brewer, K. A. Hudson-Edwards, J. Lewin, P. Scussolini, D. Eilander, A. Lechner, J. Owen, G. Bird, D. Kemp, K. R. Mangalaa, Impacts of metal mining on river systems: a global assessment. *Science (1979)* 381, 1345–1350 (2023).

16. IEA (International Energy Agency), Global Critical Minerals Outlook 2024. [Preprint] (2024). https://www.iea.org/reports/global-critical-minerals-outlook-2024.

17. S. Jasansky, M. Lieber, S. Giljum, V. Maus, An open database on global coal and metal mine production. *Sci Data* 10, 52 (2023).

18. Global Energy Monitor, Global Coal Mine Tracker, April 2024 release (2025). https://globalenergymonitor.org/projects/global-coal-mine-tracker/.

19. P. Massicotte, A. South, rnaturalearth: World Map Data from Natural Earth. R Package version 1.0.1. *Cran*, doi: https://doi.org/10.32614/CRAN.package.rnaturalearth (2023).
